# Morphometry of Concepcion Bank: Evidence of Geological and Biological Processes on a Large Volcanic Seamount of the Canary Islands Seamount Province

**DOI:** 10.1371/journal.pone.0156337

**Published:** 2016-05-31

**Authors:** Jesus Rivera, Miquel Canals, Galderic Lastras, Nuria Hermida, David Amblas, Beatriz Arrese, Pablo Martín-Sosa, Juan Acosta

**Affiliations:** 1 Instituto Español de Oceanografía, Corazón de María 8, Madrid E-28002, Spain; 2 GRC Geociències Marines, Universitat de Barcelona, Barcelona E-08028, Spain; 3 Instituto Español de Oceanografía, Vía Espaldón, dársena pesquera, Parcela 8, Santa Cruz de Tenerife E-38180, Spain; Centro de Investigacion Cientifica y Educacion Superior de Ensenada, MEXICO

## Abstract

Concepcion Bank is the largest seamount in the Canary Islands Seamount Province (CISP), an oceanic area off NW Africa including 16 main seamounts, the Canaries archipelago and the Selvagens subarchipelago. The Bank is located 90 km northeast of Lanzarote Island and has been identified as a candidate Marine Protected Area (MPA) to be included in the Natura 2000 network. A compilation of complementary datasets consisting of multibeam bathymetry, TOPAS seismic reflection profiles, side scan sonar sonographs, Remotely Operated Vehicle video records and seafloor samples allowed describing in detail and ground truthing the submarine landforms and bioconstructions exhibited by the bank. The Concepcion Bank presently rises up to 2,433 m above the adjacent seafloor and exhibits two main domains: an extensive summit plateau and steep flanks. The sub-round summit plateau is 50km by 45 km and ranges from 158 to 1,485 m depth. The steep flanks that bound it descend to depths ranging between 1,700 and 2,500 m and define a seamount base that is 66km by 53 km. This morphology is the result of constructive and erosive processes involving different time scales, volumes of material and rates of change. The volcanic emplacement phase probably lasted 25–30 million years and was likely responsible for most of the 2,730 km^3^ of material that presently form the seamount. Subsequently, marine abrasion and, possibly, subaerial erosion modulated by global sea level oscillations, levelled the formerly emerging seamount summit plateau, in particular its shallower (<400 m), flatter (<0.5°) eastern half. Subsidence associated to the crustal cooling that followed the emplacement phase further contributed the current depth range of the seamount. The deeper and steeper (2.3°) western half of Concepcion Bank may result from tectonic tilting normal to a NNE-SSW fracture line. This fracture may still be expressed on the seafloor surface at some scarps detected on the seamount’s summit. Sediment waves and cold-water coral (CWC) mounds on the bank summit plateau are the youngest features contributing to its final shaping, and may be indicative of internal wave effects. Numerous submarine canyons generally less than 10 km in length are incised on the bank’s flanks. The most developed, hierarchized canyon system runs southwest of the bank, where it merges with other canyons coming from the southern bulges attached to some sections of the seamount flanks. These bulges are postulated as having an intrusive origin, as no major headwall landslide scars have been detected and their role as deposition areas for the submarine canyons seems to be minor. The results presented document how geological processes in the past and recent to subrecent oceanographic conditions and associated active processes determined the current physiography, morphology and sedimentary patterns of Concepcion Bank, including the development and decline of CWC mounds The setting of the seamount in the regional crustal structure is also discussed.

## Introduction

Seamounts are classically viewed as “isolated seafloor features”, whose ecological and geological relevance is nowadays widely recognised (e.g. [1–3]). Seamounts often represent a sort of “island” or “seafloor nunataks” bringing heterogeneity to vast, uniform ocean expanses. As such they have been shown to have an effect on biodiversity, including living resources, and ocean circulation [4–7]. From a geological perspective, their distribution and lithology also help to explain the evolution of ocean basins and the continental margins bounding them.

Several attempts have been made to define, classify and identify the numerous seamounts in the worlds oceans (e.g. [5,8]), most of which revolve around three main concepts: size, shape and isolation. Amongst other seafloor isolated positive relieves, which are more subdued or lower in height such as hills or knolls, strictly speaking a seamount is “an elevation of the seafloor, 1,000 m or higher, either flat-topped (called a guyot or table mount) or peaked (called a seapeak)” [9]. Seamounts may be either discrete, arranged in a linear or random grouping, or connected at their bases and aligned along a ridge or rise [10].

The term “bank” that is applied to Concepcion Bank is defined by the International Hydrographic Office (IHO) as “an isolated (or group of) elevation(s) of the sea floor, over which the depth of water is relatively shallow, but sufficient for safe surface navigation” [10]. Above all, this term is a navigation concept that does not refer to a minimum elevation (e.g. 1,000 m) with respect to the surrounding seafloor. Consequently, only some “banks” are also “seamounts” but most seamounts cannot either be considered “banks” as their summit is to deep. Our target feature falls under both designations, as it respects both the geomorphological and hydrographical criteria.

Seamounts are closely related to oceanic islands as they undergo several similar stages of development from their origin on the abyssal plain to, in many cases, their submergence due to isostasy. This is why oceanic islands and seamounts often occur in association. An oceanic island can be viewed as a tall seamount in its middle age, and a guyot like an old seamount that underwent truncation by surf erosion. A knoll with a few hundred meters high can represent a young growing seamount or a partially buried old one [11]. These considerations moved some authors to propose a less restrictive height of 100 m for seamounts allowing including young and old seamounts [12–14]. The concept of isolation is included in the previous definitions and criteria, but very often seamounts are also referred to as sets of entities using terms like *group*, *seamount chain*, *ridge* or *province* [10] to express the fact that seamounts tend to concentrate in specific settings. Seamounts are more frequent on oceanic crust but appear also on transitional crust. It is relatively common that seamounts form alignments or clusters by themselves, in particular nearby spreading mid-ocean ridges, over upwelling mantle plumes or in island-arc convergence zones, thus illustrating their close genetic relation with deep geological structures and processes [15–18].

Satellite altimetry has given us the ability to identify large seamounts (namely those higher than 1,000 m) on a global scale, including those located in the most remote oceanic regions. The world seamount population is currently estimated at 171,864 seamounts [18,19]. While substantial progress on the knowledge of seamounts has been made in recent years [15], highlighting the complex geological, physical, chemical and biological processes in which they are involved, seamounts still are considered “the least explored major morphological features on Earth” [11]. This is mainly due to their large number as well as their often remote oceanic locations. Therefore, detailed studies of seamounts using modern exploration, observation and sampling technologies in particular are needed in order to deepen the understanding of the processes shaping these seafloor features [20–24]. The present paper aims to contribute to this goal by focusing on a large seamount for which a detailed morphometric analysis is performed and the driving processes are interpreted.

## Study Area

### Geological setting

**Fig 1:**
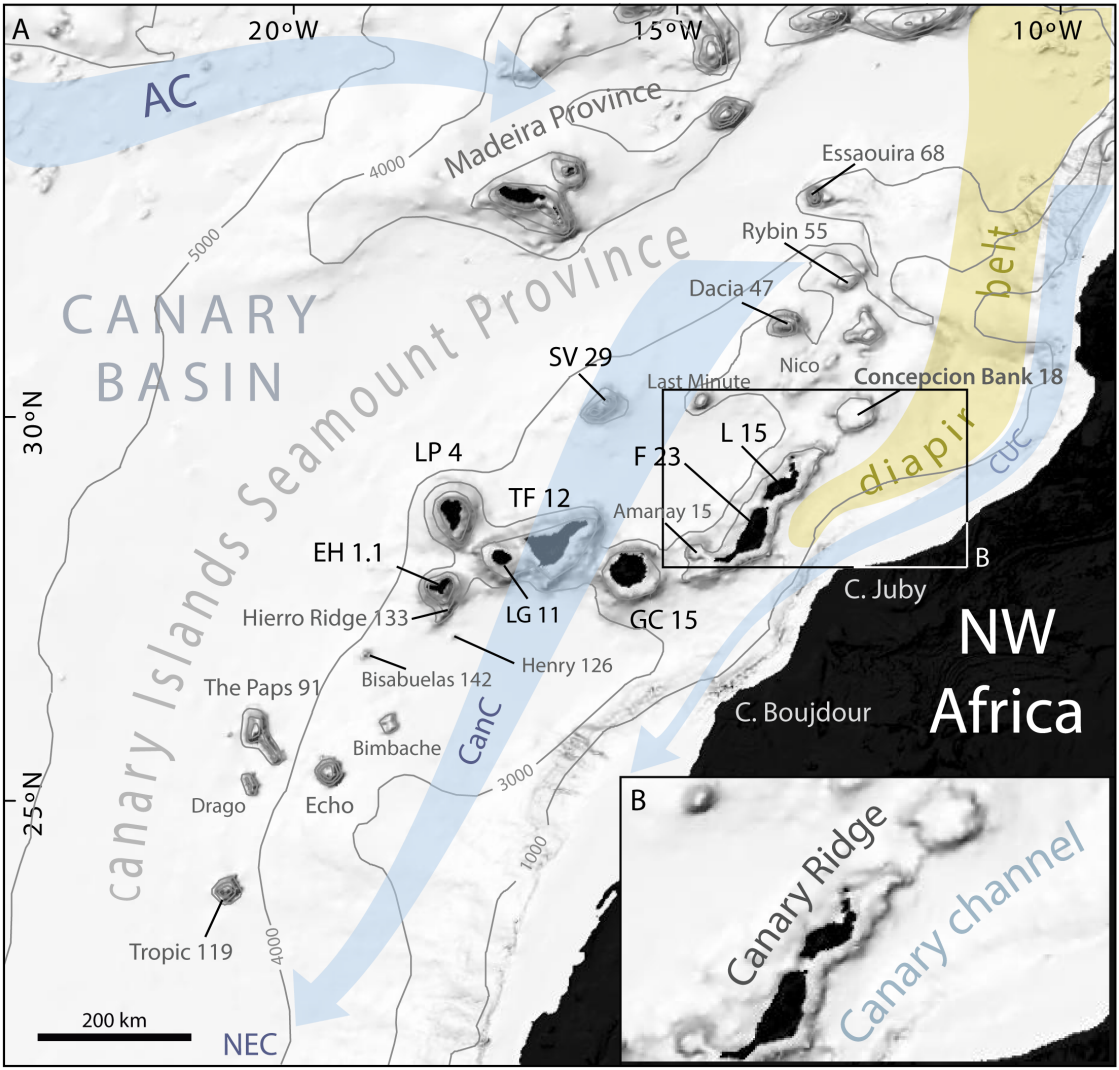
General bathymetric map of the Canary Islands Seamount Province (CISP). Note the seamounts, islands and other relevant physiographic features as well as the regional surface current system. The Canary Basin, the Canary Ridge, the Canary Channel and its diapir belt extending northwards are indicated. Numbers attached to seamounts and islands represent individual oldest age estimate where available, after Van den Bogaard [28]. AC: Azores Current. CC: Canary Current. CUC: Canary Upwelling Current. NEC: North EquatorialCurrent. SV: Selvagens. L: Lanzarote. F: Fuerteventura. GC: Gran Canaria. TF: Tenerife. LP: La Palma. EH: El Hierro. Bathymetry in meters from GEBCO.

Of the total population of seamounts taller than 1,000 m, 23,754 occur in the Atlantic Ocean, with the highest density of these features located between parallels 40°N and 55°N in the NE Atlantic [18,25]. The Canary Islands Seamount Province (CISP) is located south of this latitudinal range, in between 23°N and 33°N. It is comprised of the Canary Archipelago with 7 major islands, the two Selvagens Islets and 16 main seamounts distributed throughout an area of about 540,000 km^2^ roughly parallel to the continental margin off NW Africa (Fig 1). In addition to their geographical distribution, all these entities share clear geochemical similarities as pointed out by Geldmacher et al. [26,27]. Three main segments or alignments can be recognised along the CISP: (i) a 450 km long southern SW-NE oriented segment extending from the Tropic Seamount to the westernmost point of the Canary Islands; (ii) a 400 km long central segment roughly oriented WNW-ESE from La Palma and El Hierro islands to Fuerteventura Island; and (iii) a 500 km long northern segment, also known as the Canary Ridge, with a SW-NE oriented southern half from Fuerteventura Islands to Concepcion Bank and a S-N oriented northern part from nearby Concepcion Bank to Essaouira Seamount (Fig 1). The Selvagens Islands and the Last Minute Seamount are separated from the three main alignments and occupy a central position west of the broad crescent-shaped alignment defined by the Canary Islands and the Canary Ridge (Fig 1). The total cumulative length of the seamount and oceanic islands segments forming the CISP is therefore 1,350 km.

The features forming the CISP built up on three different crustal domains across the continental rise of NW Africa: (i) continental, (ii) transitional and (iii) oceanic crust, from east to west [29–32]. Crustal thickness decreases westwards from the continental shelf to the Canary Basin, whereas sedimentary thickness increases eastwards [30,31]. Such a gradient and the resultant isostatic balance, makes the Moho depth increase eastwards from 12 km below El Hierro Island to 20 km beneath the Canary Ridge [29].

The Concepcion Bank, along with the islands of Fuerteventura and Lanzarote, are located on the Canary Ridge (Fig 1). This is a remarkable high gravimetric gradient structure known as the Lanzarote and Fuerteventura Gradient Zone [33] that marks the limit between transitional and oceanic crust [33,34]. The Canary Ridge consists of a Tertiary volcanic core capped by the products of subsequent eruptions mainly Miocene in age [28,32,35,36]. Another prominent feature is the Canary Channel, opening between the Canary Ridge and the inner continental margin of NW Africa (Fig 1). The sediment cover in the Canary Channel is 10 km thick and ranges in age from Triassic to Present [30,31].

Evaporite deposits extend northward from the Canary Channel along a continental margin parallel belt (Fig 1). Seismic reflection profiles show early Triassic evaporites forming diapirs that intrude Mesozoic sedimentary units [30,31], occasionally reaching the seabed along the channel [30,31,37]. The diapir belt has attracted the interest of the oil industry which recently performed exploratory drillings in the area. Diapirs are absent west of the Canary Ridge where sediment thickness is much lower (i.e. up to 800 m) [29,31,38]. Another difference between the domains to the east and west of the Canary Ridge is igneous intrusions, which are very common west of the ridge and lacking east of it. Except for the main ones (i.e., those forming the modern islands, islets and seamounts), most of the structures resulting from such intrusions are nowadays covered and smoothed by sediments [35].

Of the seamounts in the Canary Ridge, Concepcion Bank (158 m), Dacia (400 m) and Essaouira (900 m) seamounts are completely or partly flat-topped, whereas Nico, Rybin and the Last Minute Seamounts are not. Nico presents two summits at 300 m and 400 m depth, and Rybin shallowest point is at 412 m. The summit of Last Minute is at 1,100 m depth. The Selvagens Islands are low-lying and rise from a couple of flat-topped structures as well [30].

In addition of being the shallowest, Concepcion Bank is also the largest seamount of the CISP. Previous studies showed that its core consists of a 10 km thick layer with a velocity of 5.6 km/s described as massive basalt [32]. This layer is covered by a thin sedimentary sequence topping pillow basalts reaching 5 to 6 km in thickness near the bank’s eastern flank [30]. The magnetic signal of Concepcion Bank shows an east to west gradient that has been interpreted as indicative of a structure with its basement kernel shifted to the west and covered by a low magnetic susceptibility volcano-sedimentary sequence increasing in thickness eastwards [39].

The origin of the Canary Islands and the CISP as a whole has been swinging between two main hypothesis: tectonics and mantle plumes [40]. The tectonic hypothesis relates the distribution of volcanic edifices to the main margin-parallel and conjugate fault systems in the region, a view that seems to be supported by the above described three main segments forming the CISP [41].

Alternatively, a mantle hotspot model has been suggested [42–44]. According to this model, volcanic edifices in the CISP should be aligned following tectonic plate drift direction and, therefore, arranged in accordance to their ages, with the western-most edifices being the youngest [45]. Long quiescence periods among eruptive events is the most important argument held by the detractors of the hotspot theory as the time gap between two eruptive episodes in the same location can last up to 40 Ma [26]. Therefore, volcanism induced from the upper mantle seems random, a view that is also supported by the distribution of historical eruption events (i.e. Lanzarote, 1824; Tenerife, 1909; La Palma, 1949, 1971; El Hierro, 2011). King and Ritsema [16] further elaborated the hotspot hypothesis, trying to overcome its weaknesses. They proposed a model describing the theoretical heat interaction between the thin oceanic crust and the thick African craton in the region, resulting in a small-scale convection cell in the upper mantle that drifts next to the craton. This model is consistent with a loose pattern of magma injection controlled by a fractured oceanic/transition crust over a sort of upper mantle bubbling area.

The recent dating of seamount rock samples has reinforced the concept of spatial randomness and long-term quiescence periods. For instance, the Bisabuelas Seamount, SW of El Hierro Island (Fig 1), is 142 Ma old whereas El Hierro is only 1.1 Ma old [32]. Furthermore, off the southern tip of El Hierro Island and adjacent to it, is a 132 Ma age ridge [28]. Interestingly, the last eruption in the Canary Islands took place in 2011–12 in between this ridge and El Hierro Island [46]. Such available ages create a puzzling situation as far as an overall age progression for the CISP goes.

### Oceanographic setting

The CISP is located inside the Canary Current System (CCS), which corresponds to the eastern section of the clockwise North Atlantic subtropical gyre, also known as the Eastern Boundary Current. The Azores Current flowing from the NW bounds the CCS to the N, while the SW flowing North Equatorial Current represents the southward extension of the CCS (Fig 1). When reaching the Canary Archipelago, the surface current of the North Atlantic subtropical gyre splits into two roughly parallel southward flowing branches: the Canary Current passing through the islands, and the Canary Upwelling Current that flows close to the African coast (Fig 1). The later induces the high primary production of the area, which supports a major fishery [47,48]. The CCS is driven by trade winds and a complex geostrophic balance resulting in a significant seasonal variability [49] also affected by the migration of the Azores high-pressure cell [50].

From top to bottom, the water column structure consists of four water masses, which are the surface water (SW), the North Atlantic Central Water (NACW), the Antarctic Intermediate Water (AAIW) and the North Atlantic Deep Water (NADW). SW is influenced by local atmospheric conditions and its lower boundary corresponds to the seasonal thermocline usually located at a depth of about 150 m [51]. Underneath the seasonal thermocline there is the NACW, a rather homogeneous water mass reaching 600 m depth and eventually more [51–54]. Below is the AAIW, which is strongly influenced, particularly in its lower levels, by the Mediterranean Water (MW) coming from the north and often forming persistent eddies called “meddies” [55–57]. This AAIW-MW layer extends down to about 1600 m. The deepest water mass is the NADW that extends from that depth down to the seabed [53]. Generally speaking, the motion of the upper water column, including the SW and the NACW is driven by trade winds. Instead, the main driving force of the deeper layers is the geostrophic balance. Between the Canary Ridge and the continental shelf (the Canary Channel) the highest velocities (>20 cm/s) have been recorded in the upper 600 m of the southward flow described above [58]. Inside this passage, a weaker poleward counter current (up to 10 cm/s) is known to exist underneath, in the AAIW depth range [59]. The boundary between these two opposite flows is at 27.3 kg m-3 neutral density that corresponds to 600 m depth throughout the region [59].

Seamounts in general, and those of the CISP in particular, are obstacles that interfere and alter the flowing of oceanic currents in different ways. For instance, White and Mohn [4] report the following ones: increase of current modulus, generation of vorticity cells with the formation of Taylor columns, baroclinic instabilities, vertical mixing, internal wave generation and resonant excitation of seamount-trapped waves through tidal forcing. These seamount-induced perturbations tend to enhance nutrient fluxes, thus leading to the development of habitats that are richer and more complex than those in the surroundings [60]. Such flow disturbance processes may also leave a morphosedimentary imprint on the seamounts.

## Data Set and Methodology

From 2009 to 2012 the InstitutoEspañol de Oceanografía (IEO) conducted five successive research cruises in order to study Concepcion Bank from a multidisciplinary viewpoint in the frame of the INDEMARES CONCEPCION Life+ project. The Ministry of Economy and Competitiveness commissioned the IEO to comprehensively characterize the seamount in order to establish its ecological significance as a candidate Marine Protected Area (MPA). Given the morphometric focus of this paper (see Introduction) the most relevant datasets used were a geophysical compilation comprising multibeam bathymetry, sub-bottom seismic reflection profiles and side scan sonar sonographs, which were supported by direct sampling and trawled camera and ROV footage for groundtruthing purposes. This data compilation allowed the first detailed geomorphic study and seafloor characterization of Concepcion Bank.

Table 1 summarizes information on the vessels, multibeam bathymetry echosoundersand sub-bottom profiling systems applied in the study of Concepcion Bank. Regular sound velocity profiles and real-time sound velocity surface measurements were performed for sound refraction correction. Vessel speed and beam spacing of the multibeam sonar were adjusted to every particular depth range in order to obtain a sounding density as homogeneous as possible (equidistant mode setting). The surveyed area was systematically covered with a 50% overlap among adjacent swaths, and eventually more in certain locations, also insonifying specific seafloor targets in different directions to improve the backscatter record. The total area covered by the multibeam data is 3,673.88 km2. Bathymetry and backscatter data were processed using CARISand Fledermaussoftware, applying post-processing refraction coefficients where needed.

**Table 1:** Multibeam bathymetry and sub-bottom profiling systems mounted onboard the three vessels used to survey the Concepcion Bank.

ResearchVessel	MBES	MBESfreq.(kHz)	SBP	SBPfreq.(kHz)
R/VVizcondedeEza	EM300	30	TOPASPS18	15–21
R/VMiguelOliver	EM302	30	TOPASPS18	15–21
R/VAngelesAlvariño	EM710	70–100	TOPASPS18	15–21

MBES: Multibeam echo-sounder. SBP: Sub-bottom profiler.

The sub-bottom profiling system was a TOPAS PS18 in all cruises. This is a very high-resolution parametric sub-bottom profiler that uses non-linear interactions between two frequencies around 18 kHz in order to achieve a narrow emission beam in the 0.5 to 6 kHz range. Penetrations up to 50 m were achieved on the Concepcion Bank. The total length of TOPAS PS18 profiles was 2,590 km. Profiles acquired over the summit plateau and deep seafloor areas surrounding the bank were highly successful. Contrastingly, those collected over the flanks were rather ineffective as seafloor steepness often produced bottom losses and abundant refraction hyperbolae.

Digital sonographs were acquired with a Geoacoustics double frequency (114–410 kHz) side scan sonar. In total 35.86 linear km of sonographs were obtained, covering an area of 9.5 km^2^. Data processing was carried out with SonarWiz. Individual sonographs were assembled to create georeferenced side scan sonar mosaics. Soft bottoms were sampled with a box corer and a beam trawl, while hard bottoms were sampled with rock dredges. Video transects were conducted using (i) the “Liropus 2000” ROV and a trawled sledge carrying a remotely operated Canon Legria HF R106 HD video camera and a Nikon D90 photo camera (ii). The ROV is a Super-Mohawk II model manufactured by SubAtlantic, rated up to 2,000 m depth and equipped with five video cameras: a Kongsberg OEI4365 colour camera, a Kongsberg OE14-502A HD video camera, one low light camera and two movable supplementary mini cameras to keep visual control of the vehicle critical components while in operation (http://www.eurofleets.eu/np4/329.html). Remote handling sampling gear consists of two Hydro-Lek manipulators and a slurp gun suction system.

All relevant georeferenced data derived from the different tools were integrated into a GIS project in order to facilitate the analysis and interpretation. The morphometric analysis was conducted with ArcGIS 10.1 and Fledermaus 7. Some ‘R’ script files written specifically for this study have been used in the statistical analyses and plotting.

## Results

### Overall morphometry of Concepcion Bank

**Fig 2:**
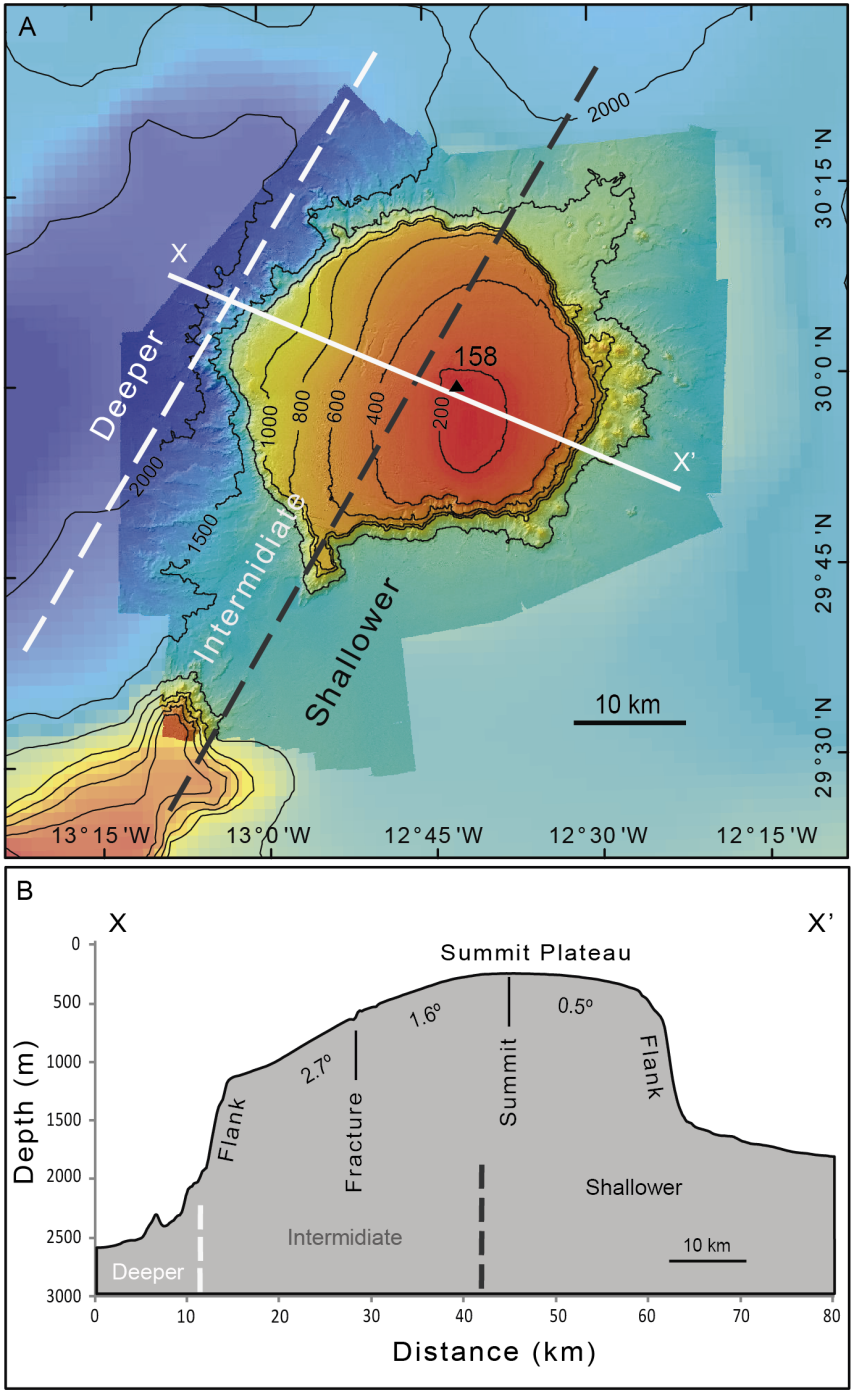
Bathymetry of Concepcion Bank and surrounding deep seafloor. (A) Bathymetric map from multibeam data (bright colours) and GEBCO (dull colours). The dashed lines delimit the three SWNW oriented parallel depth sectors described in section 4.4. (B) Bathymetric cross section along a SE-NW direction. Significant changes in slope are indicated. See location in A. Vertical exaggeration is 14:1. Summit location is indicated by a black triangle with a label showing its depth in meters.

Fig 2A shows the new high-resolution bathymetry compiled for the Concepcion Bank. It highlights the two main morphologic domains: (i) an extensive summit plateau and (ii) steep flanks surrounded by smooth abyssal seafloor. The summit plateau has a mean diameter of 48 km, an area of 1,780 km^2^ and a shallow peak rising to a minimum depth of 158m. The rim of this plateau varies in depth between 535 m and 1,485 m. Fig 2B displays a SE-NW oriented cross section of the Concepcion Bank highlighting these morphologic elements. A prominent 12 km length and up to 8 km width N-S oriented flat-topped spur, peaking at 750 m depth, occurs on the SW corner of the bank (Fig 2A). The base of the Concepcion Bank has a mean diameter of 57 km, with a shorter semi-axis of 48 km in the N-S direction and a longer 66 km semi-axis in the E-W direction.

An overall volume of 2,730 km^3^ is estimated above the seamount's 2,508 km^2^ basal surface.

**Fig 3:**
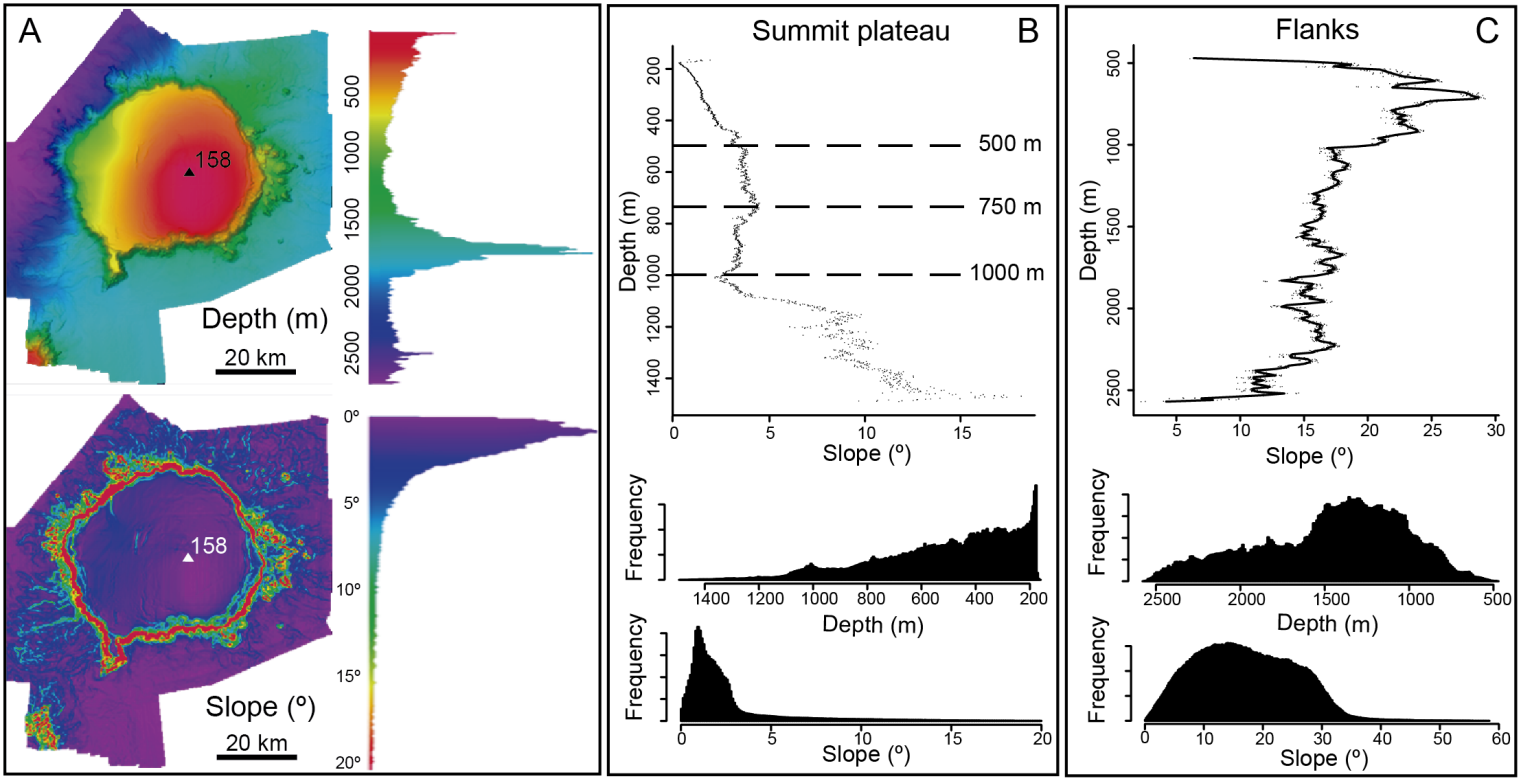
Depth and slope analysis of Concepcion Bank. (A) Depth and slope histograms. (B) Depth vs. slope plot and depth and slope histograms of the summit plateau. Dashed lines in the depth vs. slope plot indicate depths where slope changes markedly, thus helping identifying four depth ranges with distinct slope gradients and trends (158–500 m increasing, 500–750 m fairly constant, 750–1,000 m slightly decreasing, beyond 1,000 m increasing). (C) Depth vs. slope plot and depth and slope histograms of the bank flanks.

The depth histogram of the study area shows the prevalence of the depth range from 1,500 to 2,000 m followed by the depth range from 158 to 500 m. This reflects (i) the dominance of continental rise depths over which Concepcion Bank occurs and (ii) the noticeable extent of the area shallower than 500 m depth of the summit plateau (Fig 3A). The slope histogram of the study area shows the prevalence of slopes close to 2° and less than 5°, which again is due to the large areal extent of both the summit plateau and the deeper seafloor area surrounding the base of the bank (Fig 3A). The relatively small projected area taken up by the Bank flanks and the deepest area west of the flank results in lower densities for depth ranges from 500 to 1500 m and deeper than 2,000 m. Also the small size of the area covered by the bank flanks yields lower densities for slope gradients steeper than 5° (Fig 3A). This pattern becomes even more obvious when considering only the Concepcion Bank without the surrounding abyssal seafloor.

**Fig 4:**
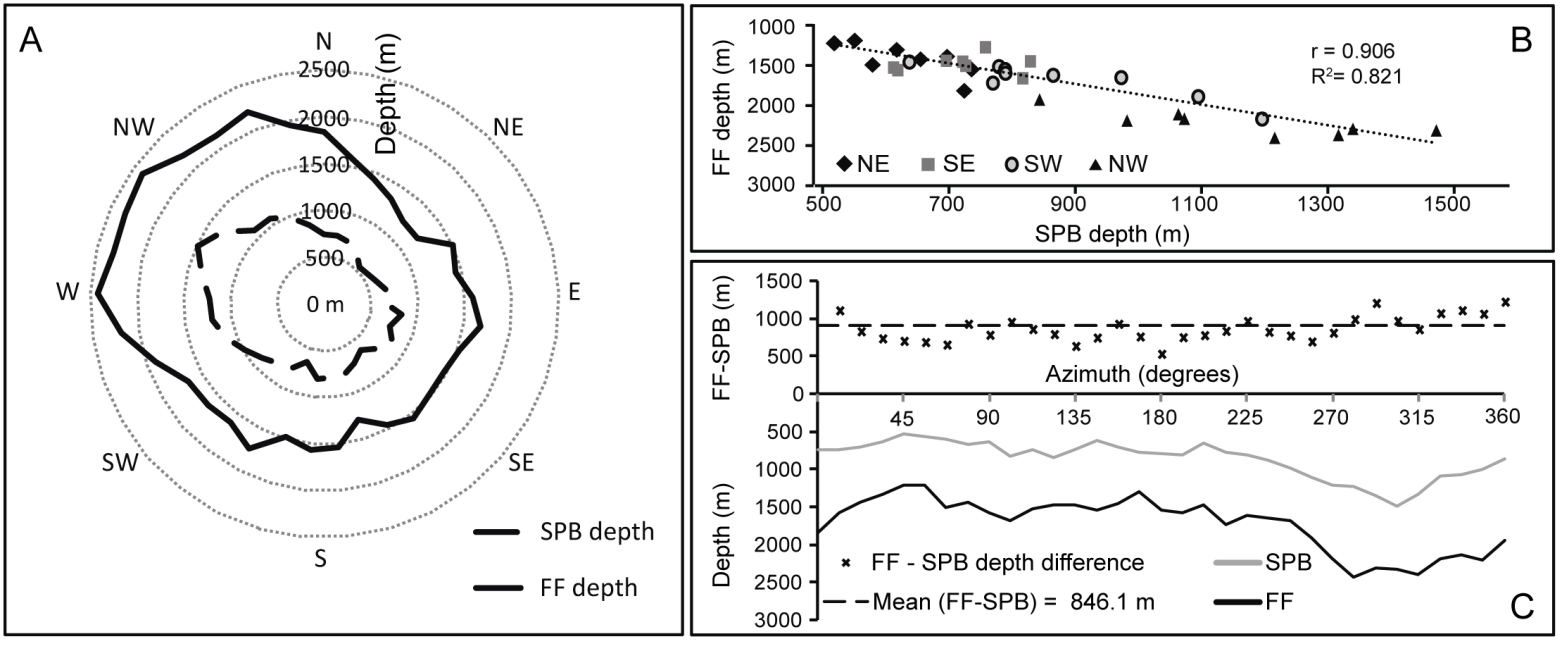
Relationship between the depth of the summit plateau slope break (SPB) and the depth of the flank foot (FF) at Concepcion Bank. (A) Polar histogram showing the SPB depth (dashed line) vs. FF depth (continuous line). (B) Correlation plot between SPB depth and FF depth per geographic sector (NE to NW). (C) Above: Height difference between FF depth and SPB depth (crosses) and the mean value of this difference (dashed line). Below: SPB and FF depths around the bank.

The basal depth of Concepcion Bank varies considerably. It is approximately 1,600 m to the E and 2,600 m on the W, yielding an longitudinal-wise basal height difference of 1,000 m (Figs 2 and 4). This is due to the location of the Bank on the continental rise, which is tilted west to north-west. In a latitudinal-wise direction the basal depth difference is much smaller (200 m) as the bank northern base lies at 1800 m and the southern one at 1600 m (Fig 4), thus reflecting the closeness of such direction to the general bathymetric trend of the continental rise.

**Fig 5:**
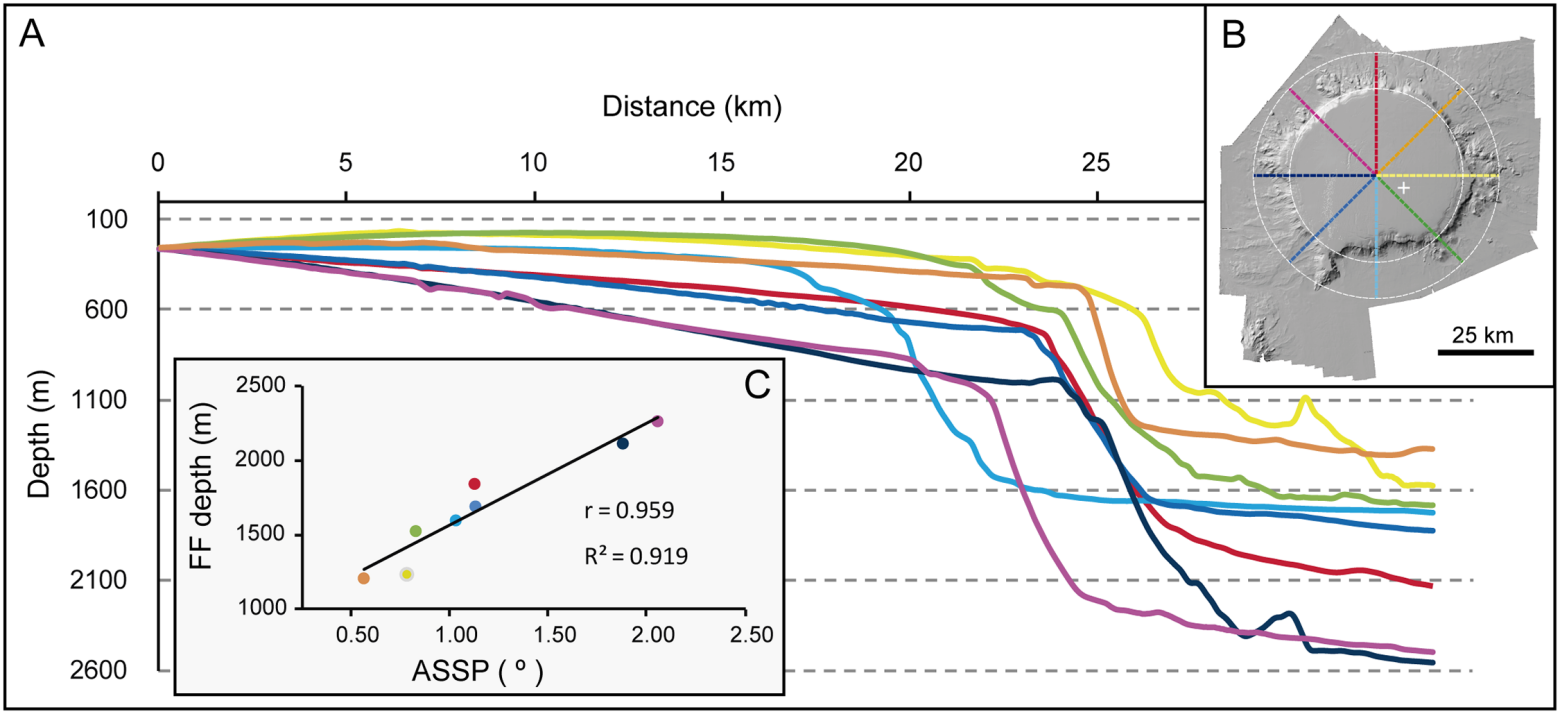
Bathymetric sections across Concepcion Bank and relationship between depth of the flank foot and slope of the summit plateau. (A) Radial depth sections from the geometric centre of Concepción Bank outwards. Note the pronounced basal height difference between the west and northwest sectors (dark blue and magenta) and the rest of the bank. Vertical exaggeration is 5:1. (B) Shaded relief image shows the location of the depth sections in A. (C) Relationship between flank foot (FF) depth and average slope of the summit plateau (ASSP) for each depth section. Colours as in A and B. Also see Table 2.

The radial depth profiles from Concepcion Bank centroid in Fig 5 are also illustrative of the morphometric variations in different directions. Variations in parameters such as the slope of the summit plateau and flanks, and the depths of the summit plateau slope break and the foot of bank flanks are highlighted (Table 2). Two main depth profile clusters emerge: (i) those heading from NE to S (orange and light blue in Fig 5A), and (ii) those heading from west to NW (dark blue and magenta), with the one heading north (red) and the one heading SW (medium blue) representing an intermediate situation. The high correlation between depth of flank foot and average slope of the summit plateau inferred by Pearson correlation coefficient (r = 0.959) should be noticed too (Fig 5C).

**Table 2:** Slope and depth values of the summit plateau, flanks and surrounding deep seafloor of Concepcion Bank according to the depth profiles in Fig 5.

Section	ASSP (°)	Std. ASSP	DSPB (m)	ASF(°)	Std. ASF	DFF (m)	ASSF(°)	Std. ASSF
North	1.13	0.72	726	16.06	5.69	1843	2.50	1.68
Northeast	0.57	1.34	507	28.35	7.96	1207	1.24	2.38
East	0.78	1.89	621	7.15	11.62	1238	6.21	6.13
Southeast	0.83	2.24	611	13.51	5.91	1525	1.46	2.77
South	1.03	1.96	615	13.67	8.52	1597	0.69	0.81
Southwest	1.13	0.97	713	12.72	6.61	1691	1.15	0.70
West	1.88	0.66	987	11.14	10.26	2115	3.83	8.40
Northwest	2.06	2.03	1052	17.69	10.10	2265	1.60	1.64

ASSP: Average slope of the summit plateau. Std. ASSP: Standard deviation of the slope of the summit plateau. DSPB: Depth of the summit plateau rim. ASF: Average slope of seamount flanks. Std. ASF: Standard deviation of slope of seamount flanks. DFF: Depth of flank foot. ASSF: Average slope of surrounding abyssal seafloor. Std. ASSF: Standard deviation of slope of surrounding abyssal seafloor.

**Fig 6:**
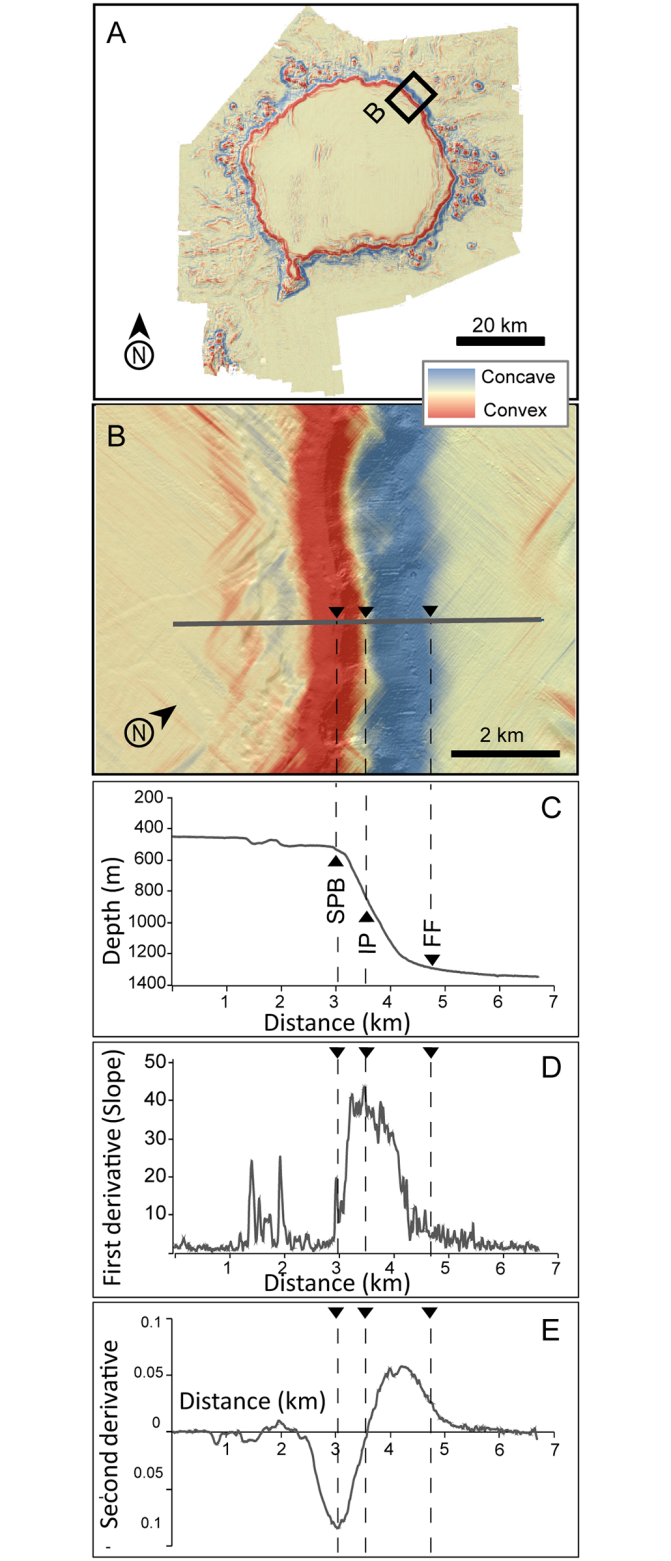
Images and plots showing how the depth of summit plateau slope break (SPB) and the depth of flank foot (FF) have been calculated. (A) Digital Terrain Model (DTM) of the along slope depth second derivative (red is negative, blue is positive). (B) Detail of the second derivative DEM across the edge of the summit plateau and foot of slope, showing a section normal to SPB and FF. An inflection point (IP) marks where the second derivative changes from convex (red) to concave (blue). (C) Depth of the section. Vertical exaggeration is 3:1. (D) First derivative (slope) along the section. (E) Second derivative (profile curvature) along the section. Dashed lines in B, C, D and E point SPB, IP and FF.

The boundaries of the three main morphometric domains in the study area (i.e. the plateau, the flanks and the abyssal seafloor surrounding the bank) have been calculated using profile curvature (the second derivative of the across slope depth). Its lowest value corresponded to the depth of summit plateau slope break, while the flank foot was delimited by a profile curvature value below 0.025 according to Heezen et al. criteria [61–62]. This is illustrated in Fig 6. A mid flank inflection point divides the convex upper section (red colour in Fig 6) from the concave lower flank (blue colour in Fig 6). At the inflection point the second derivative is zero, whereas the slope value (first derivative) is maximum (Fig 6).

### Summit plateau

The extensive summit plateau of Concepcion Bank covers one third of the surveyed area and represents 71.1% of the banks projected area. This corresponds to 2.5 times the projected area of the flanks. Although the plateau surface may look essentially flat and featureless when the overall relief of the Bank is analysed, a closer inspection reveals that it is not actually flat. The western half is noticeably tilted to the W-NW (Fig 2). This means that the slope varies with direction, with the steepest summit plateau profile corresponding to west and northwest directions and the smoothest to northeast and east directions. Sectorial and local variations in average slope are well illustrated in Fig 2C.

A westward slope increase is evident between the summit shallowest area and the western edge. The slope value varies with depth on the summit plateau, though not with the same rate for every depth interval. This is well illustrated by the slope vs. depth scatter plot in Fig 3B where different trends are shown. Between 200 and 500 m depth, a gentle depth-wise increase of the slope value is perceptible. From 500 to 750 m the slope rate increase is slightly lower and from 750 m to 1,000 m the slope is practically constant or even decreases depth-wise. In deeper areas, the slope vs. depth scatter plot does not show a clear trend due to the influence of escarpments and terraces located on the edge of the summit plateau.

The summit plateau presents a number of bedforms and morphological elements that are described below. It should be noted that the overall smoothness of the plateau facilitates the identification of low relief bedforms that could be easily missed on more abrupt terrains. This is further aided by the higher resolution of the multibeam bathymetry-derived Digital Elevation Model (DEM) at the comparatively shallower depths of the summit plateau, where the acoustic footprint is smaller than in deeper areas.

#### Sediment waves

**Fig 7:**
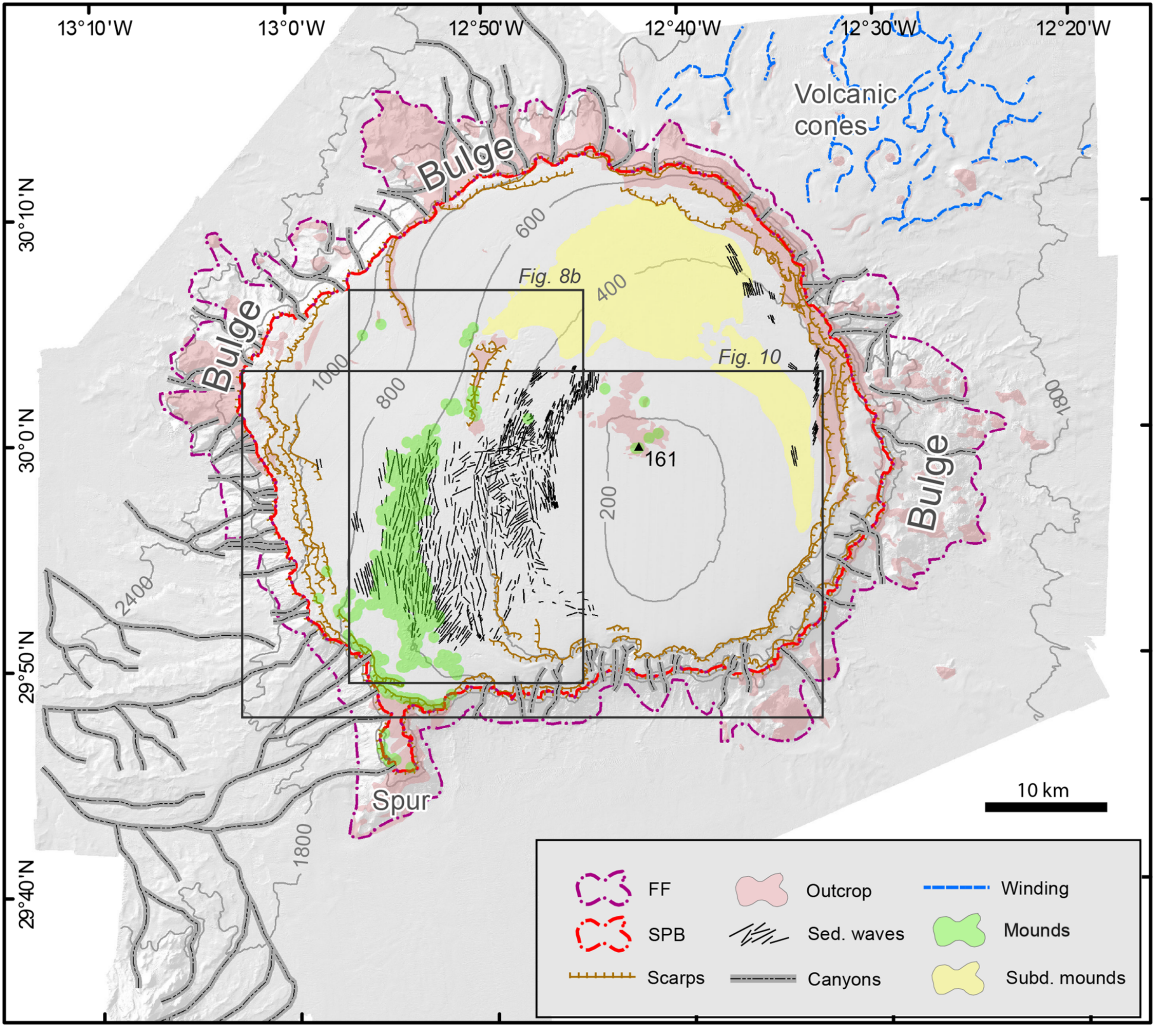
Geomorphologic features of the Concepcion Bank. The summit plateau slope break (SPB) and the flank foot (FF) separate the main morphologic domains of the bank: (i) the summit plateau, (ii) the flanks, and (iii) the surrounding abyssal seafloor.

The most prominent landforms on the summit plateau of Concepcion Bank are sediment waves forming a large field that covers an area of 300 km^2^ located on a west-facing 2° slope at the SW quadrant of the plateau (Fig 7). Furthermore, several groups of barely perceptible smooth undulations occupy an additional cumulative area of 50 km^2^ close to the northeast and east rims of the plateau, within a 200 to 400 m depth range (Fig 7).

After digitising individual sediment wave crests, an analysis of the morphometric and directional properties of sediment waves on the SW quadrant of the plateau has been performed. Length, orientation, wave amplitude (height) and wavelength were estimated. Accurate measurements in such a fine scale required special care mainly because of overlapping of wave trains with different directions and over seafloor mounds (see below). In addition, some local DEM distortions due to typical multibeam acquisition artefacts were removed or smoothed inasmuch as possible during data processing.

**Fig 8:**
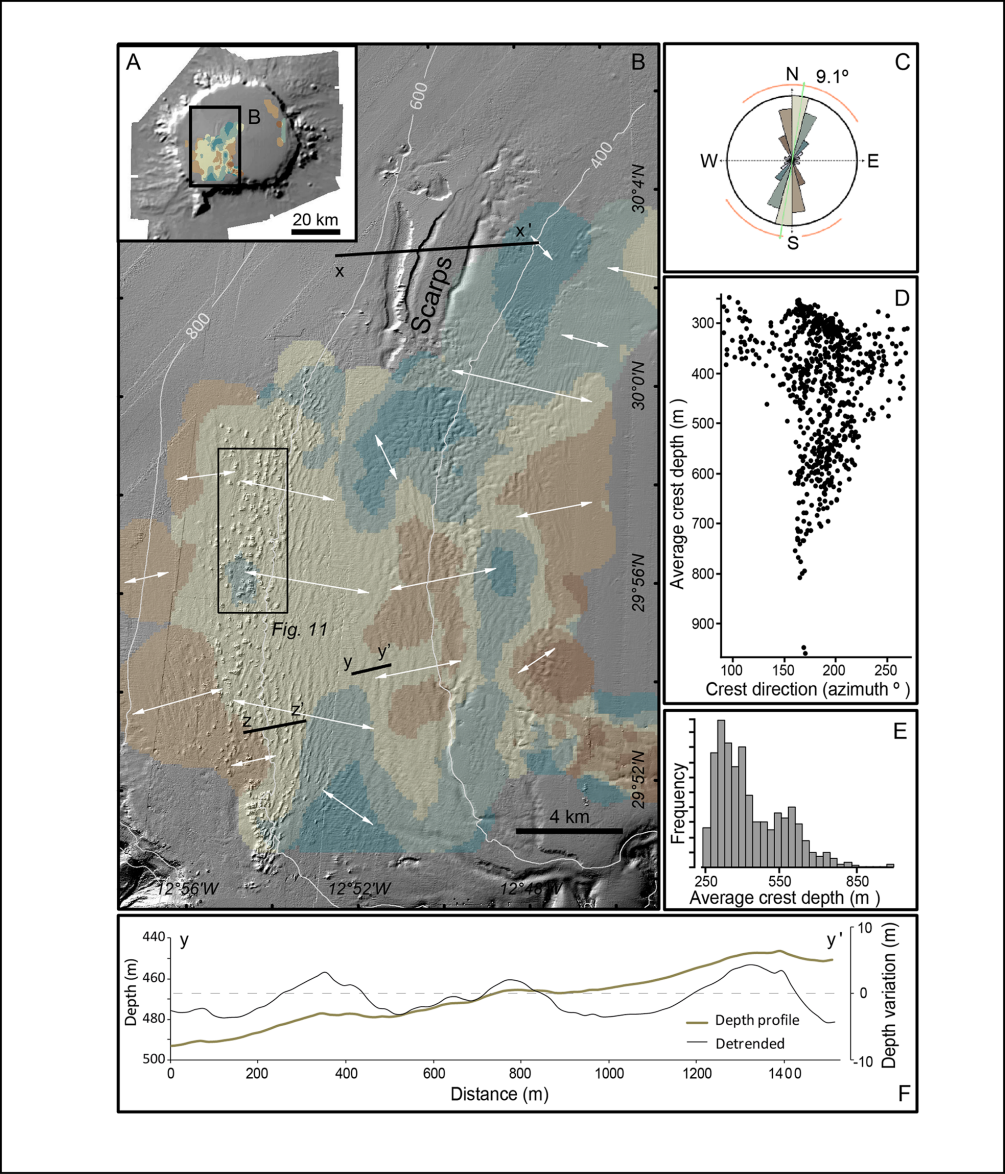
Distribution and properties of sediment waves at the Concepcion Bank summit plateau. (A) Shaded relief image showing the general distribution of sediment waves. (B) Details of the sediment wave field on the SW quadrant of the summit plateau, with colours representing different average crest directions and white arrows showing the interpreted wave migration directions. X-X’ and Z-Z’ lines locate sub-bottom profiles shown in Fig 9. Scattered isolated positive features aligned approximately NS, close to the western boundary of the sediment wave field, are mounds that are imaged in Figs 10, 11 and 12. (C) Polar diagram of the directions of sediment wave crests, with colours representing different crest directions according to B and length of sectors representing direction frequencies. (D) Crest direction vs. average crest depth plot. (E) Average crest depth frequency histogram. (F) Depth profiles across a sediment wave train. The brown solid line shows depth according to the Digital Elevation Model (DEM) of Concepcion Bank (left vertical scale), and the black solid line shows depth variation after subtracting the slope local trend along profile (detrended, right vertical axis). See location in B. Vertical exaggeration is 5:1 for the brown line and 15:1 for the black line.

The sediment waveform and distribution analysis showed that the average sediment wave is 2 to 6 m in amplitude with a 300 m wavelength. Wave crests are all oriented SSE-NNW to SSW-NNE, roughly parallel to isobaths, with a north-south (azimuth9.1°in average) wave crest orientation (Fig 8C). Variations in crest orientation allow grouping sediment waves to cluster (colours in Fig 8A, 8B and 8C). The crest orientation vs. average crest depth plot (Fig 8D) clearly shows: (i) that crest orientation is more constant with depth, and (ii)a decreasing trend in the number of distinguishable sediment waves with depths, ranging from 250m to 850m. According to this, the average crest depth frequency plot (Fig 8E) shows two modes: a dominant one at about 300m depth and a secondary one around 600m depth, which corresponds to the sediment waves with the most constant direction. Although the second mode show a lower number of sediment waves, it is worth noting that the sediment waves are larger at this depth, so comparing sediment wave abundance in terms of total length, enhances the relevance of the deeper ones.

**Fig 9:**
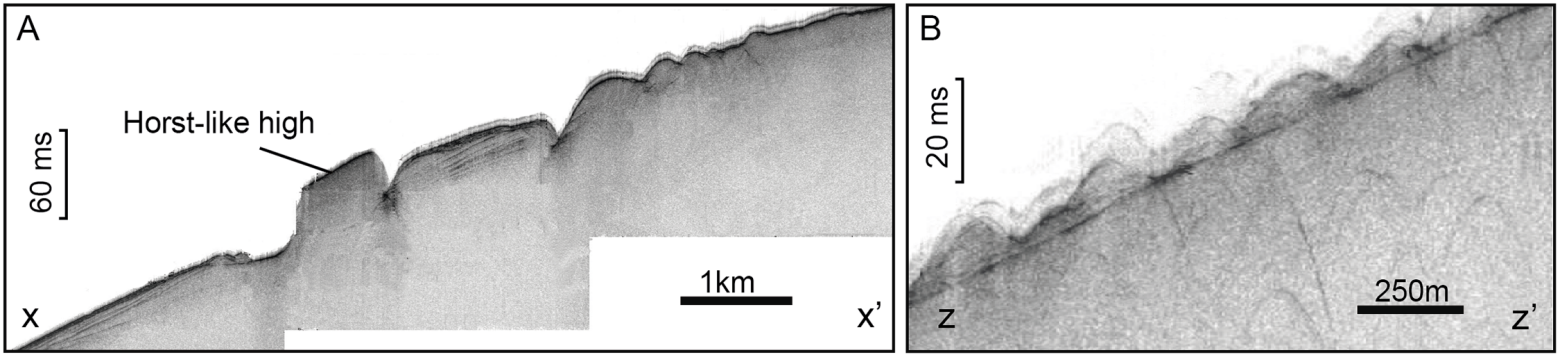
Sub-bottom seismic reflection profiles across bedforms on the summit plateau of Concepcion Bank. (A) Profile across subparallel scarps north of the sediment wave field (X-X’ in Fig 8B). These scarps mark the change in average slope indicated in Fig 2B, from 1.6° to 2.7°. Vertical exaggeration is 15:1 (Z-Z’ in Fig 8B). (B) Profile across the sediment waves interspersed with fresh-looking mounds. Vertical exaggeration is 20:1.

Cross profiles of most sediment wave trains suggests that waves are generally rather symmetrical (Fig 8F), though there are places with downslope-facing sides (e.g. up to ~3°) steeper than upslope-facing sides (e.g. ~1°) and vice-versa. Sub-bottom seismic reflection profiles show that the sediment waves interspersed by fresh-looking mounds (see further down) amidst them form a thin layer up to 15 ms TWT thick that overlies a high amplitude reflector (Fig 9A).

#### Mounds

Another noticeable morphological features on the Concepcion Bank summit plateau are mounds. Two types have been identified on distinct locations: (i) fresh-looking mounds and (ii) subdued mounds (Fig 7).

The average fresh-looking mound is round to oval shaped, with a 70 m radius and 20 m in height and an area of about 15,000 m^2^. They are mostly concentrated in an isobaths-parallel 20 km-long belt at around 600 m depth on the SW quadrant of the summit plateau, where they overlap the tallest sediment waves from the field described above. Some isolated patches of fresh-looking mounds were also observed off the main belt.

In total, the area covered by the fresh-looking mounds amounts to 120 Km^2^, of which 100 km^2^ correspond to the main belt.

**Fig 10:**
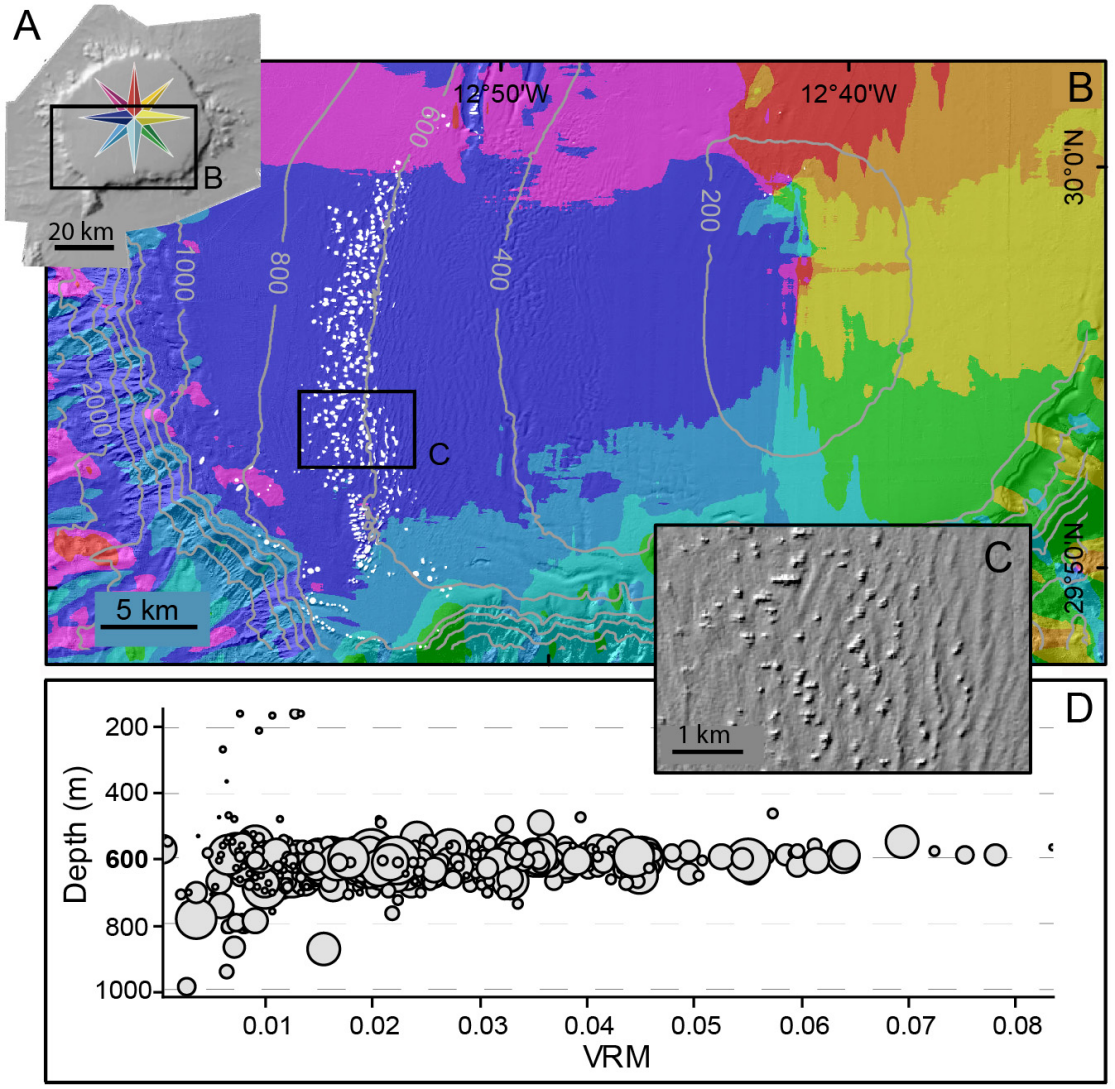
Depth-wise distribution and roughness analysis of the fresh-looking mounds on Concepcion Bank summit plateau. (A) Shaded relief of the Bank indicating area illustrated in B (color code for seafloor orientation identical to cross sections in Fig 5). (B) Seafloor orientation map of the SW quadrant of the summit plateau with white dots highlighting the location of the fresh-looking mound forming a belt of up to 4 km width, close to the 600 m isobath. Colour code in A. Location in A and Fig 7. (C) DEM detail showing scattered mounds amidst the sediment wave field. (D) Mound roughness vs. depth. Each mound is represented by a circle where diameter is proportional to mound area. Roughness has been quantified using the Vector Ruggedness Measure (VRM; Hobson [63]).

DEM roughness has been quantified using the Vector Ruggedness Measure (VRM) suggested by Hobson [63], which is defined as a function of the contiguous cells normal vector direction variance and ranges from 0 (all cells have same orientation) to 1 (complete variation). VRM for individual mounds ranged from 0 to 0.08, with most mounds (94.3%) in the interval 0–0.05, and 76.6% in the interval 0.005–0.035 (Fig 10). This means that mounds ruggedness is low in the 30 kHz multibeam records but still perceptible. No major differences in VRM were observed across the range of mound areas or depth. Independently of their area, the shallowest small and deepest larger mounds display VRM values <0.02, which means that roughness is low for deep and shallow mounds (Fig 10).

**Fig 11:**
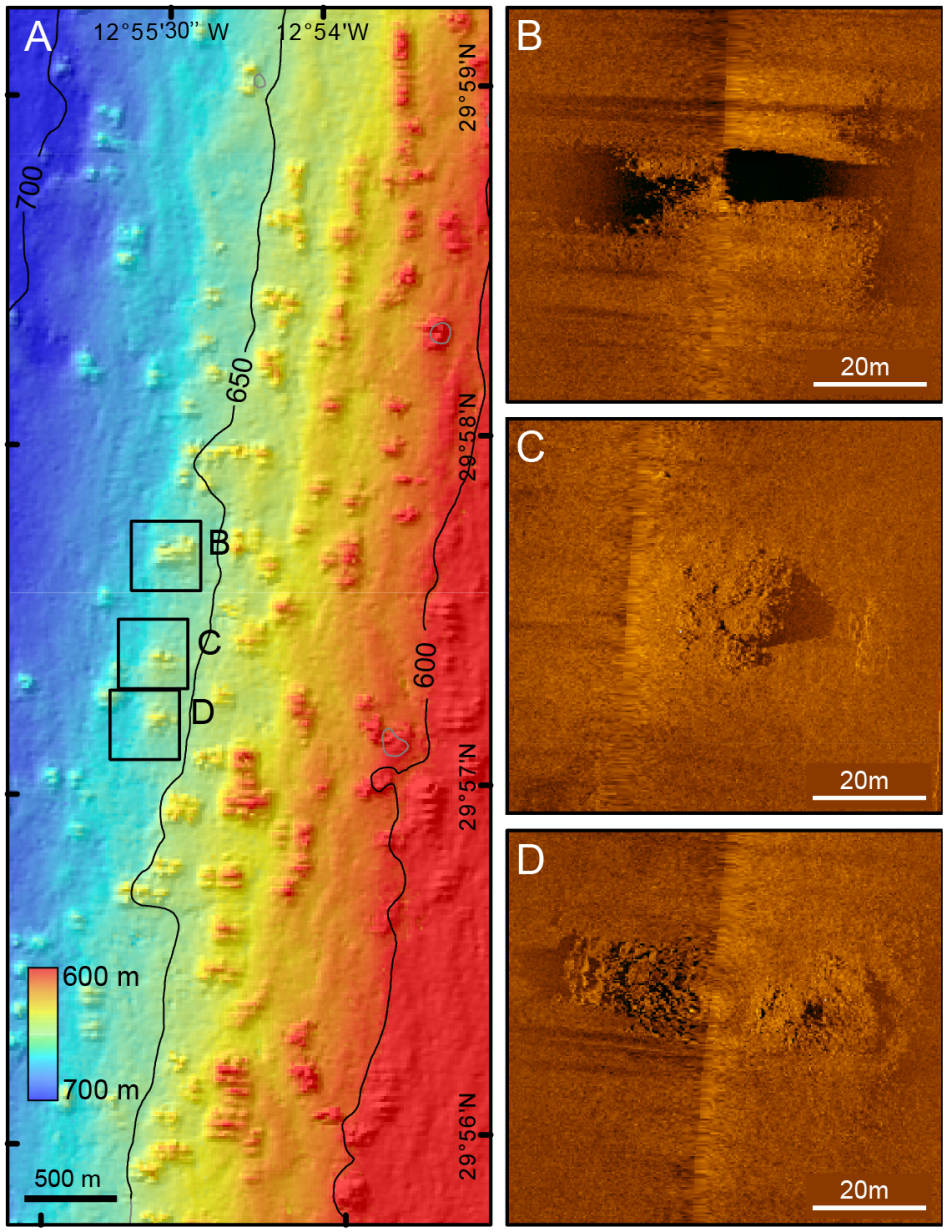
Fresh-looking mounds on the SW quadrant of the Concepcion Bank summit plateau. (A) Shaded relief image coloured by depth of mounds amidst and often on top of sediment wave crests. See location in Fig 8. (B), (C) and (D) 500 kHz side scan sonar images of three mounds. Note the higher backscatter of the rough mound tops.

The multibeam backscatter signal is stronger on top of the mounds than around them, which suggest harder mound tops. The finer scale high frequency (500 kHz) side scan sonar sonographs show an irregular surface on top of the bigger mounds but not in small ones. Some of the smaller mounds do not even exhibit high backscatter in the multibeam record (Fig 11).

Beam trawl and rock dredge samples from the fresh-looking mounds provided numerous branches of *Madrepora oculata* and *Lophelia pertusa* cold-water corals (CWC), of which dead fragments were more abundant than living ones. They also provided other species of corals and benthic organisms [64]. This allowed classification of the fresh-looking mounds of Concepcion Bank as bioconstructions. Unfortunately, the limited number of samples and the way the sampling devices work (samples are collected along more or less long strip transects over the seabed) preclude determining the precise location of living CWC colonies, both on individual mounds and over the entire mound belt.

**Fig 12:**
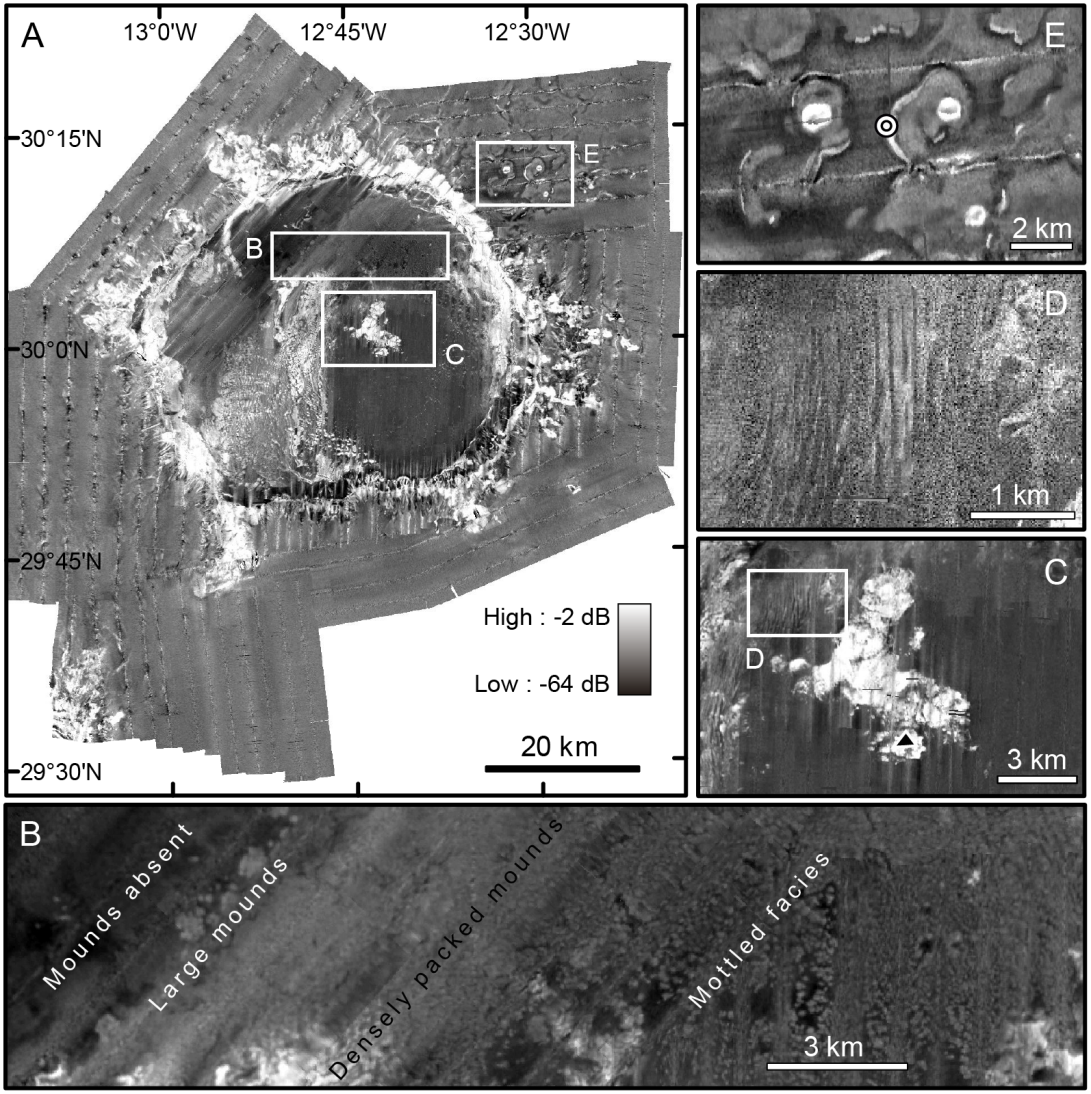
Backscatter images of Concepción Bank derived from multibeam bathymetry. High and low relative backscatter in black and white, respectively (See legend in A). (A) A general backscatter mosaic of the Bank and surrounding seafloor. Note the high backscatter of the area occupied by the sediment wave field, locally with mounds on top of the waves, on the SW part of the summit plateau (see Figs 7 and 8). The flanks of the bank mostly show high backscatter, which we mainly attribute to their steepness and presumed rocky nature. (B) Backscatter facies of the subdued mounds. Note the mottled facies to the right of the image where isolated mounds can be distinguished. A sharp backscatter shift to the left of the image marks the limit between high-density subdued mounds and an area devoid of mounds to the west. (C) Rocky outcrops on the Bank's summit; the shallowest peak is indicated by a black triangle. (D) Sediment waves with no mounds to the northwest of the rocky outcrop in C. (E) Three volcanic cones surrounded by low backscatter sediments and high backscatter moats. Small circle between the two northern cones indicate the location of pictures shown in Fig 15.

The average subdued mound has a diameter of about 100 meters, though some may reach 500 m, but they are not perceptible in the DEMas they have no three dimensional shape on the seabed. Their signature in the multibeam backscatter images is also weak but a relatively higher backscatter signal can be perceived, including a mottled pattern where individual mounds can be discriminated (Fig 12A and 12B). Where discernible, the plan shape of individual mounds is highly variable. The largest subdued mounds (>500 m in diameter) concentrate along the northwest limit of the subdued mound belt (i.e. following a straight line from the escarpments on the summit plateau to the escarpments adjacent to the plateau’s northern slope break). Immediately to the southeast, there is a 5 km wide parallel stripe where mounds seem to be smaller though they appear in such a high density that is difficult to distinguish individual entities. Mound density decreases farther to the SE, where mounds exhibit an average diameter of 100 m. No evidence of living or dead CWCs has been obtained from the subdued mound belt.

#### Scarps and central rocky outcrop

Two different sets of scarps are located on the summit plateau of Concepción Bank. Immediately to the north of the sediment wave field, three subparallelN-S to NNE-SSW are located pairs of scarps, 4 to 7 km in length and up to 100 m in height were identified (Figs 7 and 8). Apart from these, numerous rim scarps were identified roughly parallel and close to the edge of the plateau, occasionally extending towards the plateau’s interior (Fig 7). The first set of scarps resemble open cracks tapering towards their extremities until they vanish in the surrounding seafloor. A possible rotational destabilisation of the surface sediments is also observed to the east of these scarps. The location of this set of scarps coincides with a pronounced change in the average seafloor inclination, from 1.6° upslope to 2.7° downslope (Fig 9B).

**Fig 13:**
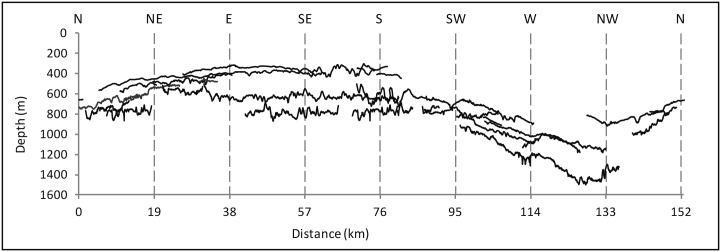
Depth profiles of rim scarps extended in a clockwise direction from N. Rim scarps are located on Fig 7. Vertical exaggeration is 20:1.

Rim scarps appear at varying depths along the edge of the plateau. In some places, up to four successive steps are visible. The height difference amongst successive scarps at a given location also is highly variable (i.e. from 10 m to 300 m). The north-starting clockwise extended plot in Fig 13 shows that these rim steps are markedly shallower from north to southwest (depth range from 320 m to 800 m), and deeper from southwest to north (600 to 1,500 m). The deepest ones (800 to 1,500 m) are in the west to northwest sector, while the shallowest ones occur in the northeast to south sector. This is obviously related to the general depth profile of the bank and, in particular, to the summit plateau, which noticeably increases depth in a westward direction (Figs 2B, 3A and 7). Single scarps can be followed for up to 50 km with a maximum depth variation of 590 m. However, in most cases such depth variation is between 10 m and 200 m (Fig 13).

An “L” shaped prominent rocky outcrop 5.5 km by 5.5 km in size and up to 30 m height occurs at about 200 m depth close to the centre of the summit plateau. The shallowest point of the seamount (158 m) is located on it (Figs 7 and 12C).

### Flanks

The summit plateau slope break, on the upper side, and the flank foot lines, on the lower side, delimit the flanks of Concepcion Bank. They cover 28.9% of the seamount area with a projected surface area of 724 km^2^. The plateau slope break ranges from 535 m to 1,485 m depth whereas the depth of the flank foot varies from 1,211 m to 2,429 m depth. Despite the wide depth ranges of both bounding lines, the height difference between both is fairly constant around a mean value of 846.1 m. This is accompanied by a high correlation (Pearson correlation coefficient = 0.906) between the depths of the summit plateau slope break and the flank foot in the different sectors of the Bank (Fig 4).

The average slope of the bank’s flanks ranges between 28.4° to the northeast and 7.2° to the east, but there is no clear spatial pattern. Fluctuations of the average slope of the bank flanks are due to the presence of bulges forming a sort of piedmonts attached to the main structure of the Bank. Such bulges result in a rough surface but a gentler average slope. The most prominent three are attached to the east, west and northwest flanks of Concepcion Bank (Fig 7). Cone-shaped features are locally observed within the flank bulges, with the main group in the larger eastern bulge that stretches 20 km in length from north to south and 12 km in width from east to west (Figs 2A and 7). The across slope profiles of Concepcion Bank flanks are close to a sigmoidal curve according to the submarine slope curvature classification of Adams and Schlager [62] However, in many sectors, a combination of the three equations proposed by the authors (Linear, Exponential and Sigmoidal), is needed in order to achieve an adequate adjustment, thus reflecting the morphological complexity of Concepcion Bank’s flanks.

**Fig 14:**
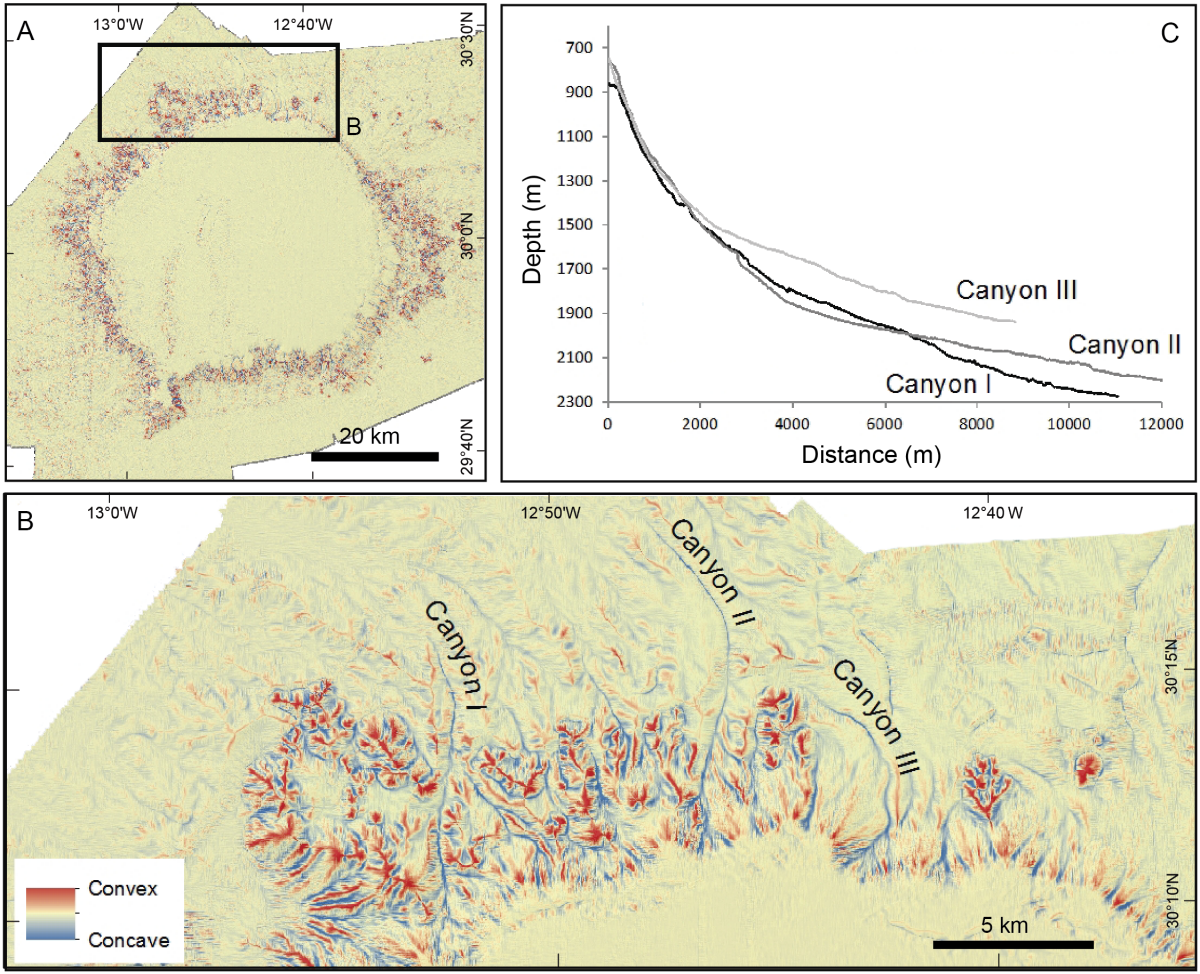
Plan curvature and characterization of submarine canyons cutting through the Concepcion Bank flanks. (A) Plan curvature map of the bank. Red colours correspond to positive plan curvature values (convex shapes) and blue to negative ones (concave shapes) highlighting crests and incisions amongst other positive and negative reliefs. (B) Detail of the plan curvature map in the northwest flank of the Bank where a submarine canyon cut into a flank-attached bulge. Coloured as in A. (C) Along-axis profiles of the three longest submarine canyons crossing the bulge attached to the northwest flank of the bank. Note differences in axial gradient between the upper and lower stretches. Vertical exaggeration is 5:1.

In addition to calculating profile curvature, we also calculated plan curvature (i.e., the second derivative of depth value normal to the slope direction) (Fig 14A). Negative values in the resulting raster identify convex surfaces indicative of ridges, knolls and other positive relief features. On the other hand, positive values identify concave features indicative of gullies, canyons, slump sidewalls and other negative relief features. This analysis highlights the main erosive morphologies along slope and, therefore, illustrates the distribution and degree of development of submarine canyons cutting through the bank flanks. In most cases, canyon axes run straight down from the summit plateau slope break to the flank’s foot on the continental rise. These straight canyons are remarkably short (3 to 5 km) and steep (15° in average). These characteristics correspond to a poor degree of canyon development, with no (or incipient) hierarchization and no (or rare)tributaries and distributaries (Fig 7). Exceptions occur where the canyons intersect flank attached bulges. Their average gentler slopes and roughness favour canyon sinuosity and length. Canyons cutting through the bulge to the northwest of the bank provide a good illustration of it (Fig 14B). These canyons extend for 9 to 12 km and have an average axial gradient of 7° with a steeper upper course (15°) and a smoother lower course (3°).

The main exception to the poorly-developed short and straight canyons is at the SW segment of the bank’s flank, including the prominent N-S oriented spur, where canyons forming a well-developed dendritic network are observed (Fig 7). Since most of the length of the canyons constituting this network is off the bank’s flank foot, we describe it in the following section on the abyssal seafloor surrounding Concepcion Bank.

### Surrounding abyssal seafloor

The extent beyond the polygon drawn by the foot of slope line represents more than 50% of the surveyed area and itis the deepest part. This section of abyssal seafloor is generally smooth and can be divided into three SW-NE oriented parallel sectors: (i) a flat (0.1° slope to the SE) shallower sector to the SE corresponding to the main mode (1,700 m) in the depth histogram (Fig 3), (ii) a flat (0.3° to the NW) deeper sector to the northwest indicated by the 2,500 m depth mode (Fig 3), and (iii) an intermediate sector accommodating the 800 m depth difference between the former two (Fig 2). This intermediate sector is rather narrow (20 km on average), slopes 2.6° north westwards and largely supports the north easternmost extension of the Canary Ridge (see Section 2.1 and Fig 1) that is Concepcion Bank. Such intermediate accommodation loses expression to the south against the Fuerteventura-Lanzarote high. In the same sector, the spur at the SW corner of Concepcion Bank also bounds the intermediate accommodation sector to the east (Fig 7).

**Fig 15:**
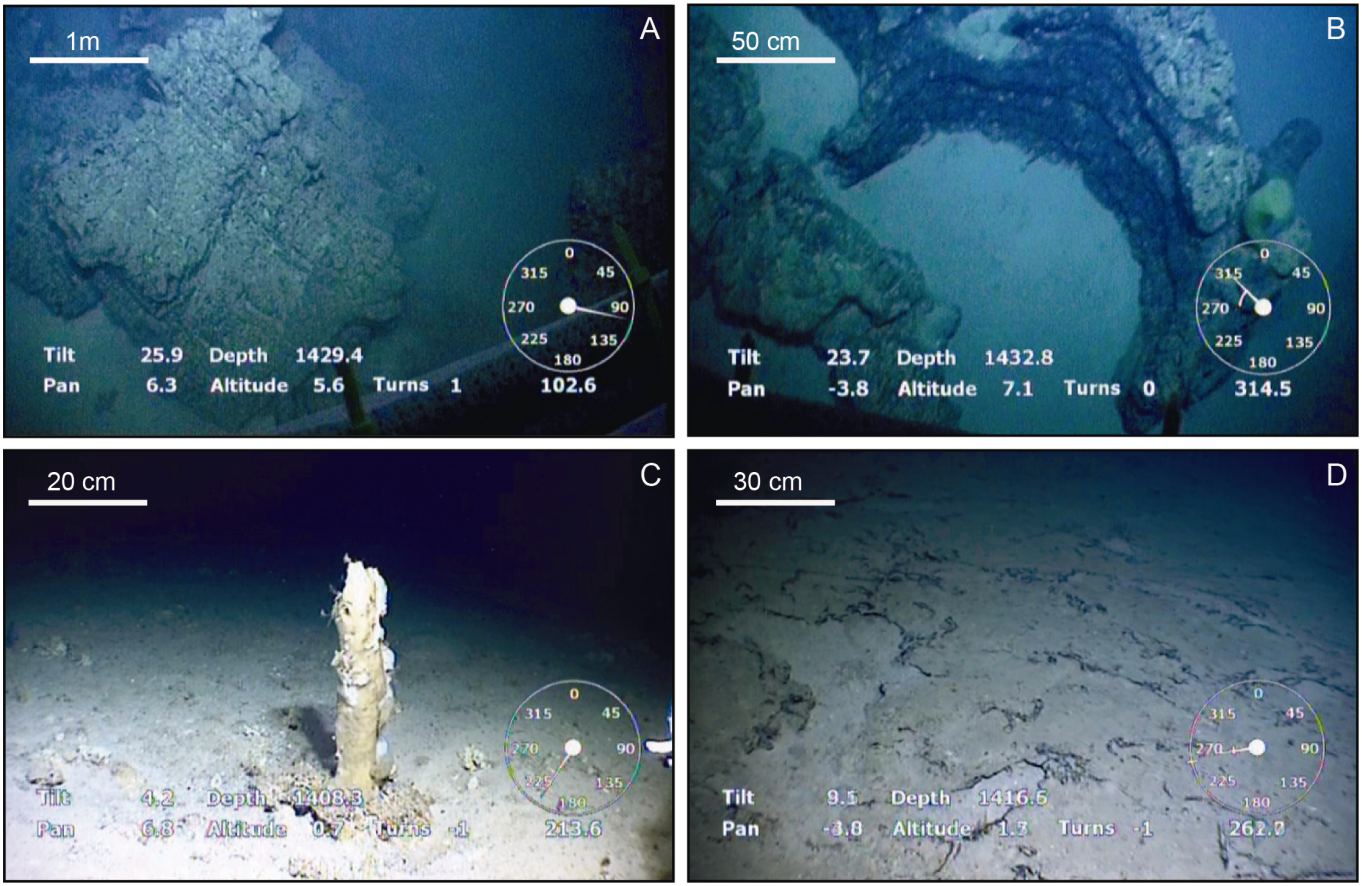
ROV seabed pictures from the northeastern part of the study area, where winding bedforms and volcanic cones co-occur (see Fig 7). (A) rocky outcrop, likely a basalt, amidst a muddy flat seafloor at 1,434 m depth. (B) Oblique view of the same rocky outcrop showing a horseshoe-shape resembling a remnant of the wall of a drained lava lobe with a collapsed upper crust. (C) 30 cm height vertical tubular shape possibly made of authigenic carbonates, surrounded by a flat seabed with scattered rock fragments at 1,409 m depth. (D) Flat seafloor with fragmented crusts on a muddy substrate at 1,418 m depth. Location of pictures is shown in Fig 12E.

The abyssal seafloor around Concepcion Bank is mostly sediment-covered, though there are local exceptions such as the area shown in Fig 12E, where rocky outcrops, seabed crusts and hard tubular structures are visible in ROV videos (Fig 15). Our high-resolution swath bathymetry data (Fig 12E), seismic profiles (S1 and S2 Figs) and video records (Fig 15) show relatively fresh volcanic cones surrounded by winding sediment bedforms northeast of the bank. Among these winding bedforms, are moats with a high backscatter signal occurrence. The high reflective signature of volcanic cones and moats in contrast to the low reflective surrounding sediments is due to the presence of bare basalts. ROV video records show seabed crusts, lava pillar-like, and other vertical tubular structures inside moats (Fig 15C and 15D).

Winding bedforms were identified only in the NE sector of the study area, in between 1,300 and 1,800 m depth. They cover about 500 km^2^ and occur in the vicinity of a few volcanic cones and other rocky outcrops (Figs 7 and 12E). These bedforms do not show any preferred orientation and are rather sinuous, occasionally anastomosing. They rise up to 80 m with respect to the adjacent seafloor and individual crests can be followed for up to 12 km (Fig 12E).

Other relatively small outcrops have also been mapped in the abyssal seafloor surrounding the bank. Erosional forms are mostly represented by the continuation of some of the submarine canyons descending from the Bank’s flanks (Fig 7). A dendritic network of submarine canyons is particularly noticeable southwest of Concepcion Bank, where courses draining the bank’s flank merge with courses coming from the Canary Channel and the flanks of the Fuerteventura-Lanzarote high to the south (Figs 2 and 7). The maximum measured length for a single course within that network was 35 km, which is likely an underestimation of its total length as some courses seem to extend beyond the study area both downslope and upslope. Within that network, the canyons cutting through the flank and base-of-flank of Concepcion Bank have more incised, V-shaped cross-sections in their steeper upper courses. As they continue downslope, they become more open in cross-sections, to the point that the seafloor expression of some of them is significantly subdued.

## Discussion

### Underlying geological control and evolution

The morphometric data presented in section 4.1 confirms the Concepcion Bank as the largest seamount of the CISP. Following the seamount classification criteria described in the Introduction, the Bank constitutes a seamount in *strictusenso*as it is an isolated elevation much taller than 1,000 m. Given its truncated profile, it can also be classified as a guyot or tablemount, although only the eastern half of its summit is actually “flat”; its western half is tilted to the WNW as illustrated by the bathymetric map in Fig 2 (see detailed description below). The fact that, in addition to Concepcion Bank, other seamounts in the Canary Ridge such as Dacia aretotally or partly flat-topped, suggesting that in the past they had reached the ocean surface, where they experienced abrasion and evolved into a guyot. Further sampling may bring new dates, 18 million years (Ma) constitutes an approximate time for Conception Bank to achieve its modern configuration. This makes Concepción Bank one of the youngest features of the northern sector of the CISP, as the nearby seamounts (Esaouira, Rybin and Dacia) are dated around 50 Ma (Fig 1). Fuerteventura (23 Ma) and Lanzarote (15 Ma) islands are closer to the Concepcion Bank age, and the three of them belong to the Canary Ridge [26,27,28].

Gravity gradients are the expression of structural discontinuities and the most remarkable one in the area is the Lanzarote-Fuerteventura Gradient Zone [33] which is aligned with the Canary Ridge. Previous geophysical studies also show that the horizontal heterogeneity of crustal velocities across the ridge increases westward whereas the Moho depth decreases in the same direction [29]. Consequently, the thickness of the crust and the sediment cover drops off westward [31]. Because of this,a significant isostatic response gradient in the east-west direction exists what means that Concepcion Bank is resting on a foundation with a westward diminishing supporting capability. This could explain the plateaus westwards tilting height drop between the summit plateau slope break and rim scarps from east to west (Fig 13) and the east-west foot of slope depth increase (Figs 2 and 4).

The contact between different crust types are favourable locations for the occurrence of fracture zones and subsequent magma intrusions. The SW-NE oriented intermediate sector (Fig 2) accommodating the 800 m depth difference between the neighbouring shallower and deeper flat seabed sectors on the deep continental rise (see section 4.4) may indicate an underlying crustal transition. An underlying fractured crust would favour magma injection [16], and could be the origin of the Canary Ridge edifices. The fact that such sector largely underlies the Concepcion Bank corroborates this view. Interestingly, the N-S oriented scarps on the central part of the summit plateau overlie this sector and may indicate a “hinge line” or flexure (Figs 2, 7, 8 and 9).

### Overimposedbedforms and bioconstructions

#### Sharp morphological entities

The slight changes in the direction of the crests and wavelengths of sediment waves forming the large field on the summit plateau (Figs 7 and 8) suggest a local variation of the driving processes or, at least, of their interaction with the plateau’s floor and the sediments on it. Two main hypotheses could help explain this sediment wave field: (i) water dynamics involving currents and/or internal waves, and (ii) downslope creep movements of the sediments.

Creeping was dismissed as a mechanism explaining the corrugated surface as sub-bottom profiles (Fig 9A) do not provide any evidence of this process, namely, deformed internal reflectors. Also, the limited sediment thickness (less than 5 m) on top of the basal high amplitude reflector (Fig 9A) and the average slope (2.7°) of the area where the sediment wave field occurs (Fig 15) seem too low for generalised creeping to occur, even if we assume that the sediment properties may allow such a type of plastic deformation. Nevertheless, localised sediment creeping may occur close to the scarps in the central part of the summit plateau (Fig 8) where some rotational destabilisation of the uppermost sediment layers is suggested (Fig 9B).

The high backscatter signature of the sediment wave field (Fig 12A) indicates that the seafloor is covered by relatively coarse sediments suggesting the occurrence of moderate to high-energy processes in this particular area. Seamounts have the capability to interfere with and divert ocean currents eventually favouring vertical mixing and creating particular structures like Taylor caps [4,65] (See also section 2.2). In addition, submarine slopes either on continental margins, seamounts or other seafloor relieves have the potential to interact with internal waves and promote their breaking, delivering energy to the seafloor.

Fresh-looking mounds with CWCs occur within the sediment wave field along a 4 km wide belt at between 570 and 710m depth (Figs 7, 8 and 10). A large majority of the mounds occur in an area of the plateau that slopes westwards (Fig 10). Most mounds lie on top of the sediment wave crests and show an elliptical shape (Fig 11). Such a position better exposes them to the incoming flows driving the sediment wave field whereasthe elliptical shape (major semi-axis perpendicular to sediment waves) could be explained by the fact that the flow-facing parts of CWC colonies have higher development rates than the less exposed ones [66,67]. These observations on sediment waves and mound patterns suggest that a west-east predominant flow impinges the seabed in the area where both features co-occur. However, no direct current measurements have been made in the area so far.

**Fig 16:**
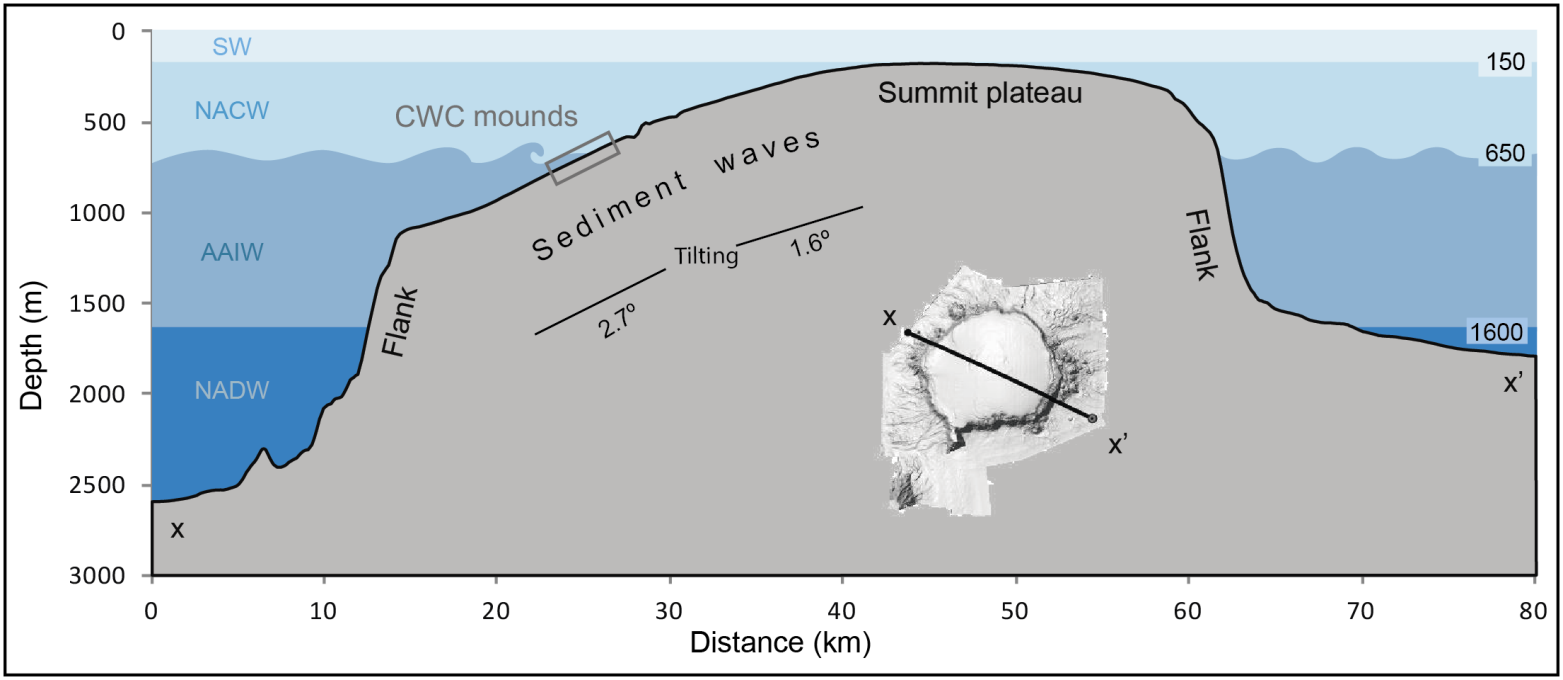
Internal waves hypothesis. Sketch illustrating the hypothesis of breaking internal waves as the main mechanism explaining the presence of the sediment wave field and the associated depth-restricted narrow belt of cold-water coral mounds on the SW sector of Conception Bank summit plateau. The bathymetric section is the same as in Fig 2B. See location in the shaded-relief DTM included in this figure.

The water depth where these two features co-occur coincides with the oscillating limit between the NACW flowing southward through the Canary Channel and the underlying AAIW flowing northward [52]. Density discontinuities and transitions, such as the one between these two water masses, are critical for the propagation of internal waves that eventually break against facing slopes. Breaking internal waves are a potentially relevant source of turbulent energy as they enable vertical mixing and particle re-suspension thus favouring coral development, as suggested by Wing [68]. The energy they deliver, mainly at the breaking zone, has also been recognised in the sedimentary record [69]. Therefore, we hypothesize that the occurrence of the sediment wave field and CWC mounds on the SW of the summit plateau is directly related to breaking waves at the interface between NACW and AAIW water masses, as illustrated in Fig 16. The mounds in particular concentrate in a narrow depth range close to 600 m depth that is where, according to our hypothesis, most energy and favourable conditions for coral growth would exist. The westward-sloping area where mounds concentrate (Fig 10) can be viewed as a sort of gentle submarine “beach” with the right orientation and inclination for breaking internal waves. The occurrence of the largest sediment waves and fresh-looking mounds along the same depth range suggests that both depend on the same process.

### Faded morphological entities

The several scattered groups of barely perceptible undulations close to the northeast and east rims of the plateau (Fig 7) are also interpreted as sediment waves. Their subdued character suggests that they may be relict or produced by weaker currents on finer sediments. These sediment waves are somehow associated to the belt of subdued mounds east and northwest of the plateau, as they occur between the edge of the belt and the plateau’s rim.

The barely perceptible relief, shape and size, and the relatively low backscatter and acoustic facies of the subdued mounds (Fig 12A and 12B) in the broad belt roughly parallel to the north-eastern and eastern edges of the summit plateau, within a depth range of 500 to 300 m (Fig 7), suggest that they are relict bioconstructions equivalent to the fresh-looking mounds in the SW corner of the plateau. The lack of dead coral branches, which are common around sub-fossil CWC mounds, indicates that these subdued mounds are quite old and that they remain detectable nowadays only because they are barely buried due to low sedimentation rates. Values of less than 10 g cm^-2^ ky^-1^ have been reported by Kuhlmann et al. [70] since the last glacial maximum. The spatial association between the subdued mounds and the smooth undulations close to the northeastern and eastern rims of the plateau reinforces the relict character of these two features. Whether living reefs on the SE sector co-existed with living mounds on the NE sector or instead CWC mound development shifted between seamount sectors is unknown at present. With or without the hypothesized shift of sediment waves and CWC mounds, what bedforms and mounds clearly show is that the energy level and associated shear stress over the bottom is nowadays much higher in the relatively steep, westward-oriented SW quadrant than in the smoother northeastern one. This observation further supports internal breaking waves at the boundary between NACW and AAIW as the main source of hydrodynamic energy over the summit plateau under present conditions.

Mound structures, both exposed and buried, similar to the ones found in Concepcion Bank, exhibiting also distinct acoustic signatures [71] have been identified in other parts of the world, namely by the numerous projects focusing on the NE Atlantic [72]. The internal structure of these mounds consists of a coral skeletal framework infilled with sediment [71]. The onset of present day living CWC mounds in the NE Atlantic has been related to the beginning of the present post-glacial period [73]. Oceanographic conditions in high latitudes during glacial periods would have been unfavourable for the development of CWC mounds while lower latitude settings, such as Conception Bank, were likely able to support such habitats also during glaciations. Eustatic sea-level fluctuations and the associated changes in hydrodynamic conditions are perhaps behind environmental shifts that regulate sediment wave activity and living conditions for the associated CWC, which eventually become relict features. It should be kept in mind that the 120 m global sea level lowstand of the Last Glacial Maximum [74] implied that the shallowest point of Concepcion Bank, without considering subsidence, was only at about 50 m below the sea surface. Whether or not the subdued and the fresh-looking CWC mounds and related sediment waves on Conception Bank represent sea level minima and maxima or different interglacial episodes remains an open question that may only be answered by drilling.

The large “L” shaped rocky outcrop close to the centre of the summit plateau (Figs 7 and 12C) constitutes a unique, single feature that stands by itself. It is detached from sediment wave and mound fields, either fresh-looking or relict, and despite it is shallower than all other mound occurrences on the plateau, some small CWC patches have been identified around and atop of it. Other guyots exhibit this type of central peak. It typically represents a residual erosional feature corresponding to the hard core of the igneous intrusion. The presence of more solid basalts offers higher resistance to marine erosion than the outer layers of volcaniclastic material originating this type of geomorphology.

### Flank processes

The best fit for the across slope profiles is achieved by the sigmoidal curve model, despite the interference of smaller scale morphological features like canyons and attached bulges. According to Adams and Schlager, “fluctuations of the wave base due to changing tides and weather or, on longer time scales, changes in sea level, interfere with the development of an exponential curvature” [62], highlighting the relevance of eustasy and subsidence on the shaping of structuresintersecting the sea surface.

#### Canyons

The submarine canyons cutting into the flanks of Concepcion Bank might have a structural control though this would be clearly different from the main SW-NE direction determining the Canary Ridge and the NW African margin, including the depth accommodation sector over which the bank stands. The length and development stage of the canyons in Concepcion Bank vary from one sector to the other. At first sight it may look as canyons are more developed where flank-attached bulges occur (i.e. northwest, north and east of the bank) and highly incipient where bulges are lacking or are poorly developed (i.e. northeast, southeast and south of the bank) (Fig 7). This could suggest a genetic relationship between bulges and canyons, perhaps related to the slope attenuation induced by the bulges that could favour canyon development, or to a bulge-feeding role of the canyons. The SW flank of the bank contradicts this view, as there is no attached bulge there and the canyon attains its maximum development (Fig 7). However the morphology of this canyon network is very different from the canyons previously referred. They are wider and longer and their cross profile is U-shaped showing a very different signature in the planar curvature DTM (Fig 14). These morphological differences should respond to different flow regimes and to the influence of the passage separating Concepcion Bank and Lanzarote Island. This passage is set on the Canary Ridge dividing the eastern and relatively shallower Canary Channel from the western and deeper abyssal seafloor (Fig 1). Seismic profiles (S5 Fig) show a chaotic facies between both domains evidencing mass wasting flows.

#### Bulges

Rocky bulges can correspond to mass wasting deposits, lava flows, igneous intrusions, or a combination of these events. The mass wasting hypothesis is unlikely, as there are no major embayments that could be interpreted as landslide scars along the perimeter of the summit plateau. In fact, two of the three main bulges attached to Concepcion Banks flanks are off smooth projections west and east of the plateau edge and the third one to the north is off a roughly straight edge (Fig 7). In contrast to the younger western Canary Islands of Tenerife, La Palma and El Hierro, where large amphitheatre-shaped headwall scars with a dramatic expression on both subaerial and submarine landslides have been identified [75–81], no similar scars occur on the older islands of Fuerteventura and Lanzarote, which are the closest to Concepcion Bank both geographically and in terms of age (Fig 1). However two evacuation areas on the submarine flanks west of the Fuerteventura-Lanzarote block have been related to large buried landslide deposits [82] located on the west side of each island. The absence of large mass wasting scars on the edge and flanks of Concepcion bank does not totally preclude the possibility that old, buried landslide deposits arising from the Canary Ridge exist, in particular on the east flank, facing the Canary Channel (Fig 1B), where geophysical studies show a 10 km thick sedimentary cover [30]. Moreover subaerial erosion of the seamount top in Miocene times could have erased or concealed the scars and debris fan of the older mass wasting events through deposition of substantial amounts of sediment. Nevertheless, volcanic cones are frequent on the island aprons of the archipelago particularly in those areas unaltered by massive landslides. They reach high densities in the Anaga offshore Massif (Tenerife island), the north flank and south rift of La Palma Island, the three rifts of El Hierro [82] and Lanzarote western slope for example. This kind of submarine volcanoes most typically present a conical shape, but other morphologies are also possible, namely elongated ridges probably built up byfissural eruptions. Their typical signature in the seismic profiles is a transparent core outlined by a sharp chevron shape reflector (S3 and S4 Figs).

#### Basal processes

The co-occurrence of winding sediment bedforms and fresh-looking volcanic cones in the same deep-water location may suggest a relationship between the two. The lack of sediments covering the volcanic cones and the rocks inside the moats suggest that they are relatively young in age or that the environment does not favour sediment deposition. The seismic profiles of the winding bedforms (S1 and S2 Figs) and their smooth shapes resemble contourite deposits induced by bottom currents flowing around obstacles [83]. However, the low velocity of bottom currents [59] and the fact that these bedforms do not show any preferred orientation but have highly sinuous shapes, occasionally merging into each other, is not in accordance with contourite currents steadily flowing along a sustained direction.

The presence of dolomite in sediments [84] is indicative, although not exclusive, of hydrothermal activity [85], Sediment samples analyzed and described by Quevedo-Gonzalez et al. [84] show that sediments from the winding bedforms contain more than 5% of dolomite and around 40% of calcite in the finest sediment fraction (<45 μm) whereas in other samples within the bank calcite represent less than the 20% weight of the fine sediment fraction and dolomite is absent [64,84]. Evidence of hydrothermal activity long after seamount emplacement has been reported in other CISP seamounts. The Cretaceous age of Henry Seamount and the occurrence of hydrothermal venting in the Holocene is an example [86].

According to previous geophysical studies [26,30] the Canary Ridge continues north-eastwards beyond Concepcion Bank in the form of a massive igneous intrusion forming a broad elevation barely covered by sediments. This elevation has a relative low magnetic amplitude signal that contrasts with a strong gravity signature. Dañobeitia suggests that this circumstance could be explained by thermal activity related to Concepcion Bank [35]. Two main high reflective seismic reflectors are present in the seismic records from this area. According to DSDP 415 the shallower reflector, at around 200 m below seafloor surface, corresponds to early-middle Miocene material and a deeper one, 475 m below the modern seabed, to late Paleocene [87]. The igneous structure underlying fresh volcanic cones and winding shapes rises from the deep reflector whereas the shallower one onlaps the structure, indicating an age between the Paleocene and the Miocene [35].

So far, any possible relation between seafloor volcanic or hydrothermal activity eventually driving near-bottom currents capable of transporting sediments cannot be neither demonstrated nor excluded without bottom current measurements over a period of time.

## Conclusions

With an age of 18 Ma, an overall volume of 2,730 km3 and a basal area of 2,508 km2, Concepcion Bank is the largest seamount in the 400 km long Canary Ridge. It stands above the continental rise of NW Africa and is dominated by a westwards tilted large summit plateau delimited by abrupt flanks leading to a surrounding deeper area. Most of the seamount stands over a margin-parallel SE-NW sector accommodating 800 m of across-margin vertical offset, from 1,700 to 2,500 m. We consider that such an accommodation is related to a crustal transition with associated fracturing. Some scarps close to the centre of the summit plateau occur directly above this depth-accommodation belt with roughly the same direction. Local faulting and magma injections according to King and Ritsema’s view [16], might help explaining the extended volcanic activity of the Canary Ridge, which culminates on the Concepcion Bank and the islands of Fuerteventura and Lanzarote. The Canary Ridge continues north-eastwards, at around 1,500m depth, exhibiting morphological features such as bare volcanic cones, lava pillar-like structures and volcanic crusts that we interpret as evidence of relatively recent volcanism [88,89]. Furthermore, the presence of dolomite within the sediments suggests some remnant hydrothermal activity. Winding bedforms identified in the same area may be driven by contour bottom currents, flows induced by hydrothermal venting or a combination of both.

Previous studies suggest that the most active processes on seamounts take place at its shallowest parts [2,4,5,60,65]. Our findings at Concepcion Bank would concur with this view and furthermore they also show a complex interaction between physical and biological processes at different time scales highlight the significance of the underlying structural geological control (See S1 Video for a general overview). According to our interpretation, the E-W, structurally determined height difference of the base of Concepcion Bank and the westward tilting of most of its summit plateau largely influence how hydrodynamic processes interact with the plateau floor where sediment wave fields and bioconstructions, both relict and active, have been found. Breaking internal waves propagating at the boundary between two water masses are the best candidate for delivering hydrodynamic energy at the depths where fresh-looking sediment waves and bioconstructions co-occur. The presence of relict bedforms and, likely, CWC mounds at specific sectors of the summit plateau suggest variations in oceanographic conditions, potentially related to glacial-interglacial oscillations, causing the deactivation of sedimentary and biological systems.

## Supporting Information

S1 Fig Seismic profile of the winding bedforms. (TIF)

S2 Fig Seismic profile of the winding bedforms. (TIF)

S3 Fig Seismic profile of the Southeast flank bulges. (TIF)

S4 Fig 3D view and bathymetric profile of the East flank bulges. (TIF)

S5 Fig Seismic profile of the transition between Canary Channel and deep seafloor. (TIF)

S1 Video Virtual flight trough Concepcion Bank. (MP4)
