# Supplementary figures and images for: Morphometry of Concepcion Bank: Evidence of Geological and Biological Processes on a Large Volcanic Seamount of the Canary Islands Seamount Province
Source: PLoS One. 2016 May 31;11(5):e0156337. doi: 10.1371/journal.pone.0156337 (PMC4886973; doi:10.1371/journal.pone.0156337)

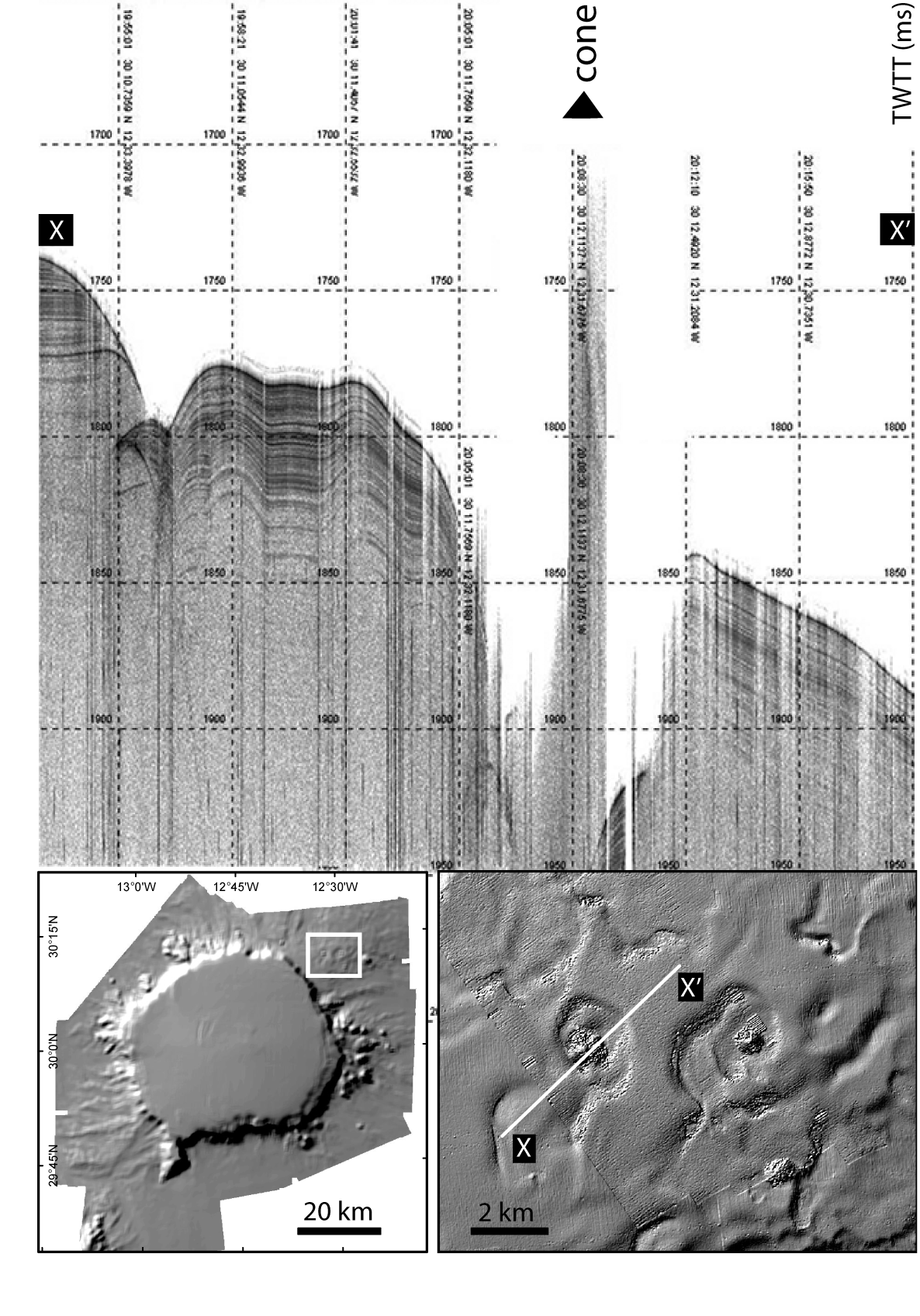

Supplement: S1 Fig — (TIF) [file pone.0156337.s001.tif]

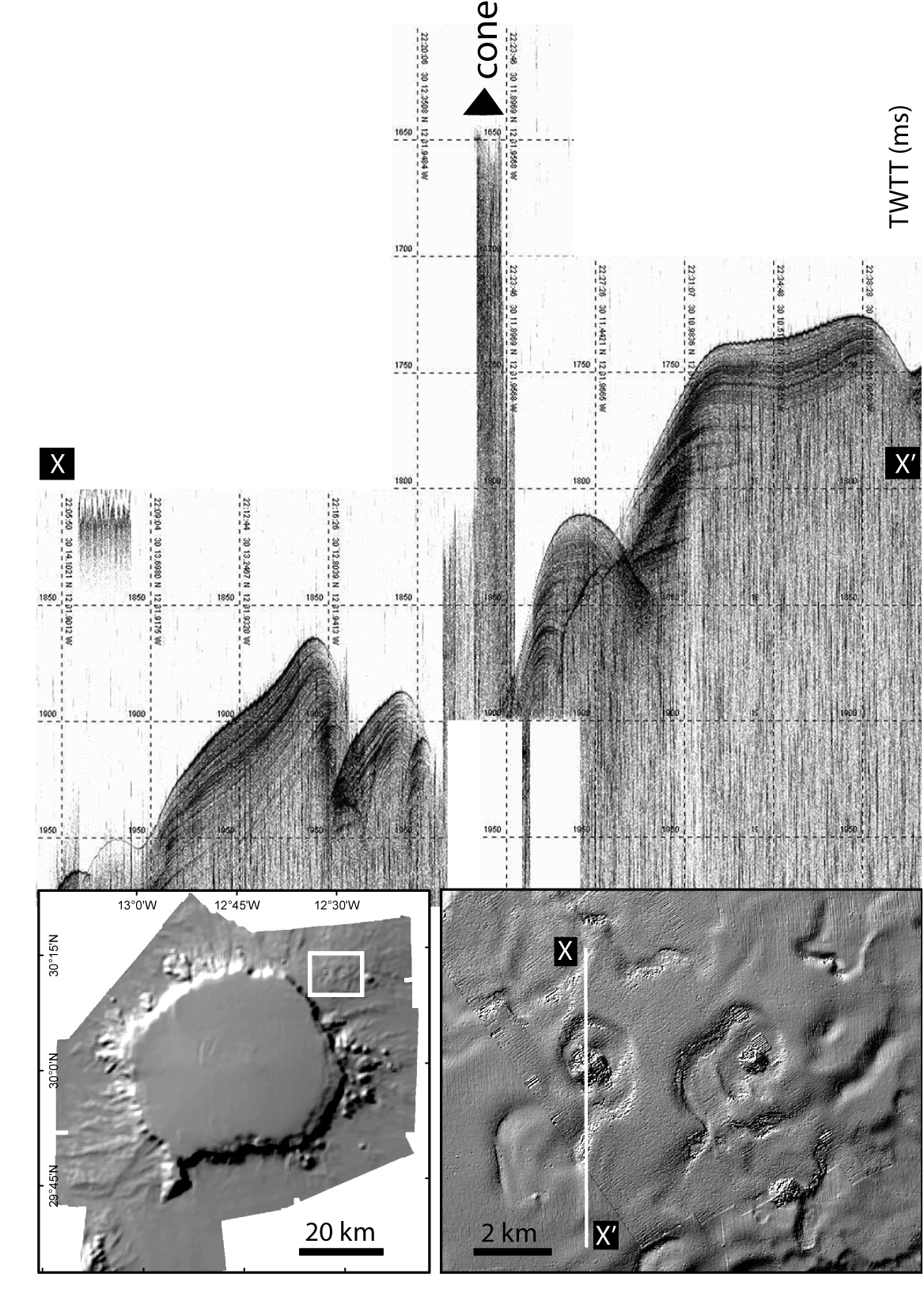

Supplement: S2 Fig — (TIF) [file pone.0156337.s002.tif]

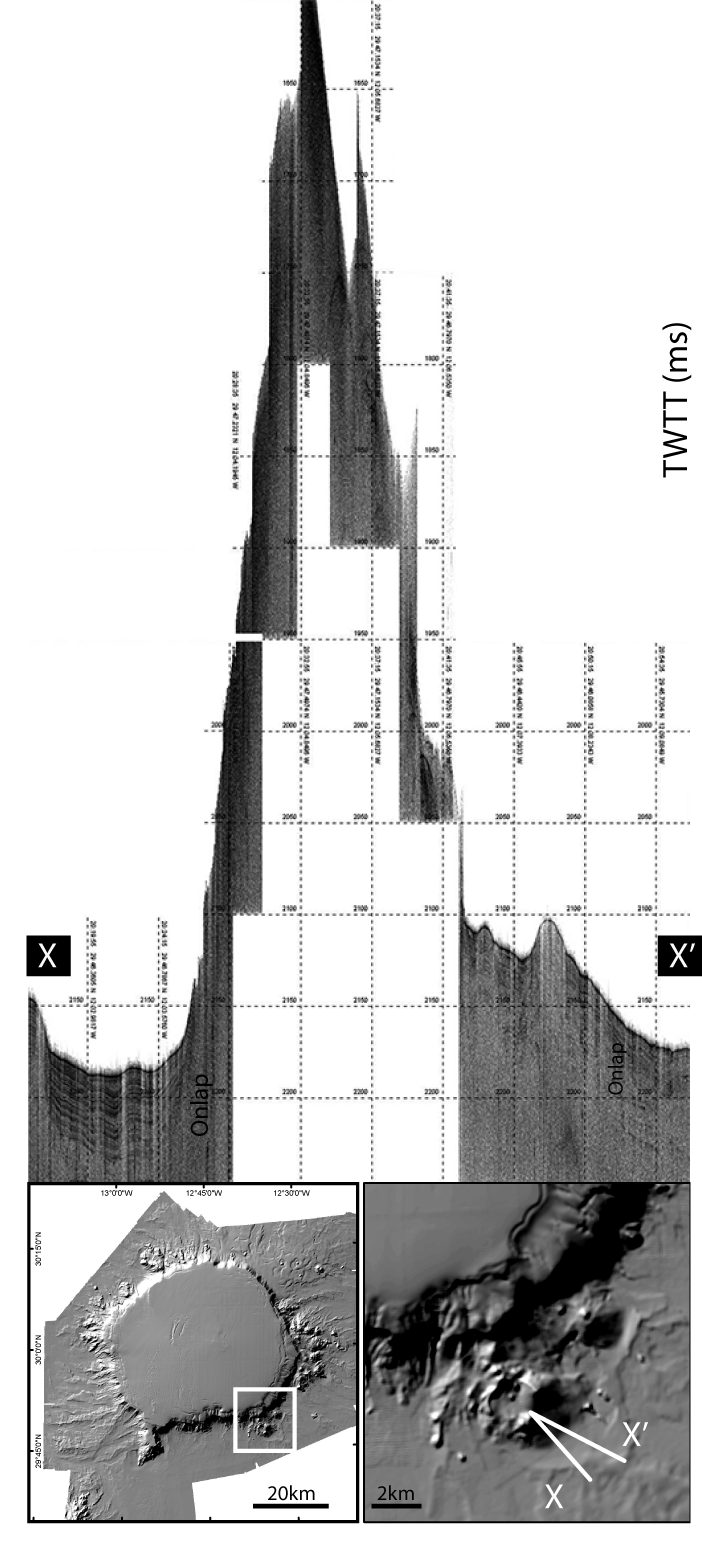

Supplement: S3 Fig — (TIF) [file pone.0156337.s003.tif]

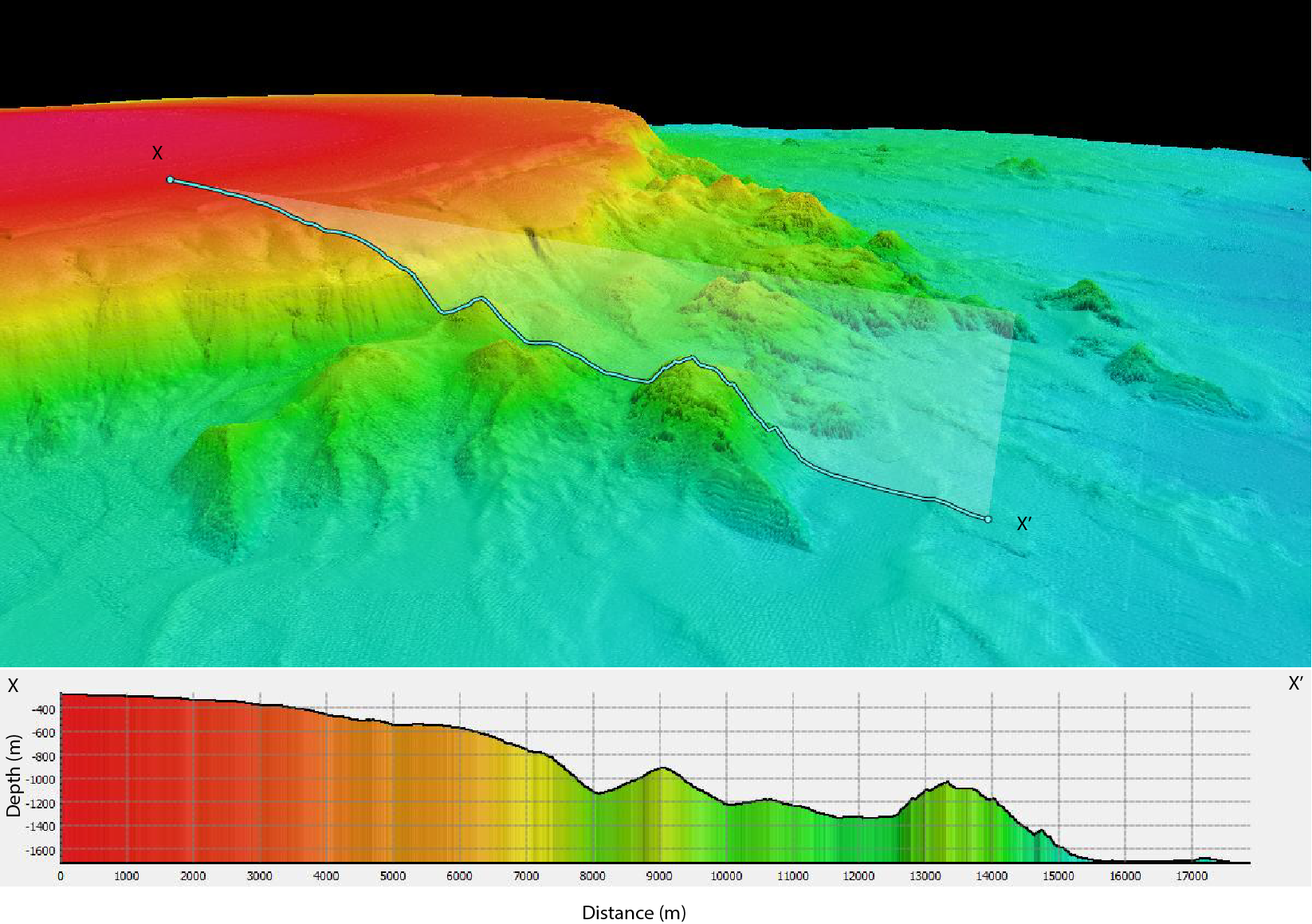

Supplement: S4 Fig — (TIF) [file pone.0156337.s004.tif]

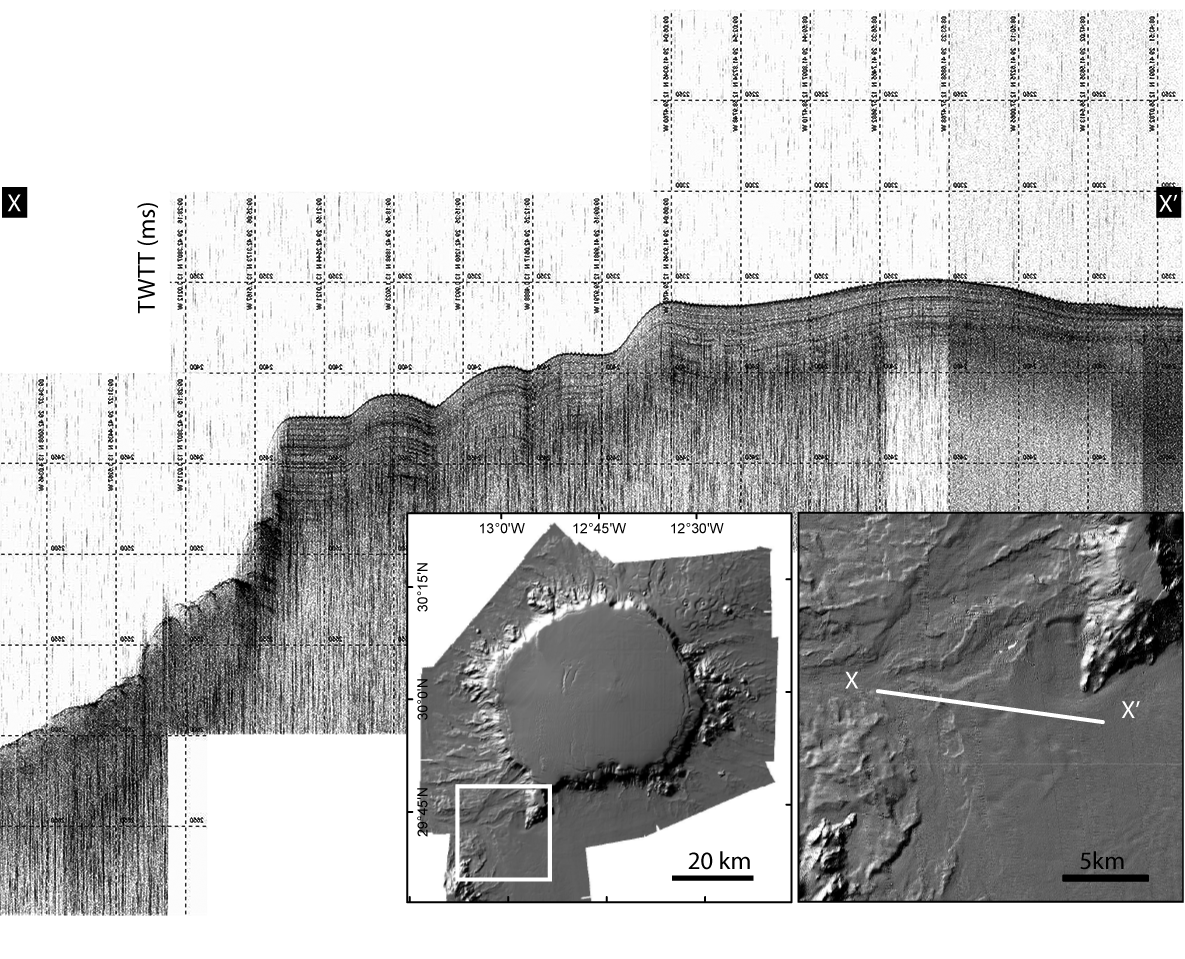

Supplement: S5 Fig — (TIF) [file pone.0156337.s005.tif]
